# The 5th edition of the World Health Organization Classification of Haematolymphoid Tumours: Myeloid and Histiocytic/Dendritic Neoplasms

**DOI:** 10.1038/s41375-022-01613-1

**Published:** 2022-06-22

**Authors:** Joseph D. Khoury, Eric Solary, Oussama Abla, Yassmine Akkari, Rita Alaggio, Jane F. Apperley, Rafael Bejar, Emilio Berti, Lambert Busque, John K. C. Chan, Weina Chen, Xueyan Chen, Wee-Joo Chng, John K. Choi, Isabel Colmenero, Sarah E. Coupland, Nicholas C. P. Cross, Daphne De Jong, M. Tarek Elghetany, Emiko Takahashi, Jean-Francois Emile, Judith Ferry, Linda Fogelstrand, Michaela Fontenay, Ulrich Germing, Sumeet Gujral, Torsten Haferlach, Claire Harrison, Jennelle C. Hodge, Shimin Hu, Joop H. Jansen, Rashmi Kanagal-Shamanna, Hagop M. Kantarjian, Christian P. Kratz, Xiao-Qiu Li, Megan S. Lim, Keith Loeb, Sanam Loghavi, Andrea Marcogliese, Soheil Meshinchi, Phillip Michaels, Kikkeri N. Naresh, Yasodha Natkunam, Reza Nejati, German Ott, Eric Padron, Keyur P. Patel, Nikhil Patkar, Jennifer Picarsic, Uwe Platzbecker, Irene Roberts, Anna Schuh, William Sewell, Reiner Siebert, Prashant Tembhare, Jeffrey Tyner, Srdan Verstovsek, Wei Wang, Brent Wood, Wenbin Xiao, Cecilia Yeung, Andreas Hochhaus

**Affiliations:** 1 Department of Hematopathology, The University of Texas MD Anderson Cancer Center, Houston, TX USA; 2 Department of Hematology, Gustave Roussy Cancer Center, Université Paris-Saclay, Villejuif, France; 3 Division of Hematology/Oncology, The Hospital for Sick Children, Toronto, ON Canada; 4 The Steve and Cindy Rasmussen Institute for Genomic Medicine, Nationwide Children’s Hospital, Columbus, OH USA; 5 Pathology Unit, Department of Laboratories, Bambino Gesu Children’s Hospital, IRCCS, Rome, Italy; 6 Centre for Haematology, Imperial College London, London, UK; 7 Moores Cancer Center, University of California San Diego, La Jolla, CA USA; 8 University of Milan, Fondazione Cà Granda, IRCCS, Ospedale Maggiore Policlinico, Milano, Italy; 9 Service d’hématologie, oncologie et transplantation, Hôpital Maisonneuve-Rosemont, Université de Montréal, Montréal, QC Canada; 10 Department of Pathology, Queen Elizabeth Hospital, Kowloon, Hong Kong; 11 Department of Pathology, The University of Texas Southwestern Medical Center, Dallas, TX USA; 12 Department of Laboratory Medicine and Pathology, University of Washington, Seattle, WA USA; 13 Department of Hematology-Oncology, National University Cancer Institute, Singapore, Singapore; 14 Department of Pathology, The University of Alabama at Birmingham, Birmingham, AL USA; 15 Department of Pathology, Hospital Infantil Universitario Niño Jesús, Madrid, Spain; 16 Liverpool Clinical Laboratories, Liverpool University Hospitals Foundation Trust, Liverpool, UK; 17 Faculty of Medicine, University of Southampton, Southampton, UK; 18 Amsterdam UMC, Location Vrije Universiteit Amsterdam, Department of Pathology, Amsterdam, The Netherlands; 19 Department of Pathology & Immunology, Baylor College of Medicine, Texas Children’s Hospital, Houston, TX USA; 20 Department of Pathology, Aichi Medical University Hospital, Nagakute, Japan; 21 Department of Pathology, Ambroise Pare Hospital, AP-HP and Versailles SQY University, Boulogne, France; 22 Department of Pathology, Massachusetts General Hospital and Harvard Medical School, Boston, MA USA; 23 Department of Laboratory Medicine, Institute of Biomedicine, Sahlgrenska Academy at University of Gothenburg and Department of Clinical Chemistry, Sahlgrenska University Hospital, Gothenburg, Sweden; 24 Laboratory of Hematology, Assistance Publique-Hôpitaux de Paris, Cochin Hospital and Université Paris Cité, CNRS, INSERM, Cochin Institute, Paris, France; 25 Department of Hematology, Oncology, and Clinical Immunology, Heinrich-Heine-University, Düsseldorf, Germany; 26 Department of Pathology, Tata Memorial Hospital, Mumbai, India; 27 MLL Munich Leukemia Laboratory, Munich, Germany; 28 Department of Haematology, Guys and St Thomas’ NHS Foundation Trust, London, UK; 29 Indiana University School of Medicine, Indianapolis, IN USA; 30 Lab Hematology, Dept LABGK, Radboud University Medical Center, Nijmegen, The Netherlands; 31 Department of Leukemia, The University of Texas MD Anderson Cancer Center, Houston, TX USA; 32 Pediatric Hematology and Oncology, Hannover Medical School, Hannover, Germany; 33 Departments of Pathology and Oncology, Fudan University, Shanghai, China; 34 Department of Pathology and Laboratory Medicine, University of Pennsylvania, Philadelphia, PA USA; 35 Section of Pathology, Clinical Research Division, Fred Hutchinson Cancer Center, Seattle, WA USA; 36 Pediatric Hematology and Oncology, Clinical Research Division, Fred Hutchinson Cancer Research Center, Seattle, WA USA; 37 Department of Pathology, Beth Israel Deaconess Medical Center, Boston, MA USA; 38 Department of Pathology, Stanford University School of Medicine, Stanford, CA USA; 39 Department of Pathology, Fox Chase Cancer Center, Philadelphia, PA USA; 40 Department of Clinical Pathology, Robert-Bosch-Krankenhaus, and Dr. Margarete Fischer-Bosch Institute of Clinical Pharmacology, Stuttgart, Germany; 41 Malignant Hematology, H. Lee Moffitt Cancer Center and Research Institute, Tampa, FL USA; 42 Hematopathology Laboratory, Tata Memorial Hospital, Mumbai, India; 43 Pathology and Lab Medicine, Cincinnati Children’s Hospital Medical Center, Cincinnati, OH USA; 44 Department of Hematology and Cellular Therapy, University Hospital Leipzig, Leipzig, Germany; 45 Department of Paediatrics, University of Oxford, Oxford, UK; 46 Department of Oncology, University of Oxford, Oxford, UK; 47 Immunology Division, Garvan Institute of Medical Research, Sydney, Australia; 48 Institute of Human Genetics, Ulm University and Ulm University Medical Center, Ulm, Germany; 49 Cell, Developmental & Cancer Biology Department, Knight Cancer Institute, Oregon Health & Science University, Portland, OR USA; 50 Department of Pathology and Laboratory Medicine, Children’s Hospital Los Angeles, Los Angeles, CA USA; 51 Department of Pathology and Laboratory Medicine, Memorial Sloan Kettering Cancer Center, New York, NY USA; 52 Hematology/Oncology, Universitätsklinikum Jena, Jena, Germany

**Keywords:** Haematological cancer, Diagnosis

## Abstract

The upcoming 5th edition of the World Health Organization (WHO) Classification of Haematolymphoid Tumours is part of an effort to hierarchically catalogue human cancers arising in various organ systems within a single relational database. This paper summarizes the new WHO classification scheme for myeloid and histiocytic/dendritic neoplasms and provides an overview of the principles and rationale underpinning changes from the prior edition. The definition and diagnosis of disease types continues to be based on multiple clinicopathologic parameters, but with refinement of diagnostic criteria and emphasis on therapeutically and/or prognostically actionable biomarkers. While a genetic basis for defining diseases is sought where possible, the classification strives to keep practical worldwide applicability in perspective. The result is an enhanced, contemporary, evidence-based classification of myeloid and histiocytic/dendritic neoplasms, rooted in molecular biology and an organizational structure that permits future scalability as new discoveries continue to inexorably inform future editions.

## Introduction

The World Health Organization (WHO) classification of tumours is an evidence-based classification of cancers occurring within various organ systems. It is a standard for diagnosis, research, cancer registries, and public health monitoring worldwide. For the first time since the inception of the classification over 60 years ago, the current series (5th edition) has been developed within a unified relational database framework that encompasses the entirety of human cancers. Tumours of each organ system and across volumes (blue books) are classified hierarchically within this novel framework along taxonomy principles and a set of non-negotiables that include process transparency, bibliographic rigor, and avoidance of bias [1, 2]. The development of the 5th edition is overseen by an editorial board that includes *standing members*—representatives from major medical and scientific organizations around the world—who oversee the entire series, in addition to *expert members* appointed for their leadership and contemporaneous expertise relevant to a particular volume [3]. The editorial board, in turn, identifies authors through an informed bibliometry process, with an emphasis on broad geographic representation and multidisciplinary expertise. By design, multidisciplinary author/editor groups (a total of 420 contributors) shared overlapping coverage of disease categories to ensure conceptual continuity and content harmonization. This approach reflects the ways in which the classification is meant to be implemented, with multidisciplinary input that emphasizes a holistic approach to patient management from diagnosis through disease monitoring.

The aim of this paper is to provide an overview of the new edition of the WHO classification for myeloid and histiocytic/dendritic tumours. The last edition of the haematolymphoid classification dates back to 2008 and was revised in 2017. An overview of the lymphoid tumours is provided in a companion manuscript [4].

The classification structure follows a lineage-based framework, flowing broadly from benign to malignant and branching down to category, family, type (disease/tumour), and subtype. Where possible, a triad of attributes was systematically applied and included: lineage + dominant clinical attribute + dominant biologic attribute. Lineage attribution rests on immunophenotyping with flow cytometry and/or immunohistochemistry. Dominant clinical attributes are general features of the untreated disease and include descriptors such as acute, chronic, cytopenia(s) (myelodysplasia) and cytosis(es) (myeloproliferation). Most biologic attributes include gene *fusions*, *rearrangements*, and *mutations*. Fusions are part of the nomenclature of types/subtypes when the identities of both implicated genes are required or often desirable criteria for diagnosis (e.g., *PML*::*RARA*). Rearrangements, a broad term that encompasses a range of structural genomic alterations leading to gene fusions, are part of the nomenclature of types/subtypes when there are multiple possible fusion partner genes of a biologically dominant gene (e.g., *KMT2A*). Of note, the use of the term rearrangements is maintained in the classification due to its wide usage across prior editions, although it is recognized that it is more appropriate for genomic modifications in genes consisting of various segments (e.g., immunoglobulin genes and T-cell receptor genes). A deliberate attempt is made to prioritize classifying tumour types based on *defining genetic abnormalities* where possible.

Emerging entities are listed as disease subtypes under a novel rubric of *other defined genetic alterations*. This is envisioned as a landing spot in the classification to incorporate new/rare entities whose recognition is increasing as high-throughput molecular diagnostic tools become more available. This approach replaces the assignment of provisional status to such entities. It is recognized that the diagnosis of such subtypes might not be feasible in all practice settings. A set of decision support guidelines was adopted to aid in determining what subtypes would qualify in this context; they include: (1) having distinct molecular or cytogenetic features driven by established oncogenic mechanisms; (2) not meeting subtype criteria under other tumour types with defining genetic abnormalities; (3) having distinct pathologic and clinical features, including - but not limited to - response to therapeutic interventions; and, (4) at least two quality peer-review publications by distinct investigator groups.

The application of this classification is predicated on integrating morphologic (cytology and histology), immunophenotypic, molecular and cytogenetic data. This is in line with previous editions, with expanded numbers of disease types and subtypes that are molecularly defined. It is hoped that the genetic underpinnings of the classification will prompt the provision of health resources to ensure that the necessary genetic testing platforms are available to peruse the full potential of the classification. Notwithstanding, the full published classification will include listing of essential diagnostic criteria that have the broadest possible applicability, particularly in limited resource settings. A further aid to broader applicability is the improved hierarchical structure of the classification, which permits reverting to family (class)-level definitions when detailed molecular genetic analyses may not be feasible; this approach is further elaborated on in the introduction of the blue book.

In line with the rest of the WHO 5th edition series, the classification of myeloid and histiocytic/dendritic neoplasms follows the Human Genome Organization Gene Nomenclature Committee recommendations, including the new designation of gene fusions using double colon marks (::) [5].

## Clonal haematopoiesis

Clonal haematopoiesis (CH) refers broadly to the presence of a population of cells derived from a mutated multipotent stem/progenitor cell harbouring a selective growth advantage in the absence of unexplained cytopenias, haematological cancers, or other clonal disorders. The incidence of CH increases with age [6]. Substantial advances in understanding the molecular genetics and public health implications of CH took place since the last classification, including recognition of their association with increased overall mortality, cardiovascular diseases, and myeloid malignancies. More specific emerging associations, such as those characterizing the VEXAS (*v*acuoles, *E*1 enzyme, *X*-linked, *a*utoinflammatory, *s*omatic *UBA1* mutations) syndrome [7], represent manifestations of the interplay between inflammation and CH/myeloid neoplasia that are being gradually uncovered. Inclusion of CH in the classification represents a key inaugural effort to define and codify such myeloid precursor lesions.

*Clonal haematopoiesis of indeterminate potential* (CHIP) is defined in the classification as a term referring specifically to CH harbouring somatic mutations of myeloid malignancy-associated genes detected in the blood or bone marrow at a variant allele fraction (VAF) of ≥ 2% (≥4% for X-linked gene mutations in males) in individuals without a diagnosed haematologic disorder or unexplained cytopenia [8]. (Supplemental Data Table S1) The significance of variants detected at lower levels is unclear at present.

*Clonal cytopenia of undetermined significance* (CCUS) is defined as CHIP detected in the presence of one or more persistent cytopenias that are otherwise unexplained by haematologic or non-haematologic conditions and that do not meet diagnostic criteria for defined myeloid neoplasms. Cytopenia definitions are harmonized for CCUS, MDS, and MDS/MPN; they include Hb <13 g/dL in males and <12 g/dL in females for anaemia, absolute neutrophil count <1.8 ×10^9^/L for leukopenia, and platelets <150 × 10^9^/L for thrombocytopenia [9].

### Summary Box:

- CH is recognized as a category of precursor myeloid disease state.
- CHIP and CCUS are formally defined.

## Myeloproliferative neoplasms

Myeloproliferative neoplasms (MPN) are listed in Table 1. The main types remain largely unchanged from the prior edition. Initial diagnostic evaluation of MPN continues to depend on close correlation between clinical features, molecular diagnostics, and usually morphologic evaluation of a trephine bone marrow biopsy. Most MPN patients are diagnosed in chronic phase (CP), which may progress into a blast phase (BP) associated with the accumulation of secondary cytogenetic and/or molecular aberrations.

**Table 1:** Myeloproliferative neoplasms.

Chronic myeloid leukaemia
Polycythaemia vera
Essential thrombocythaemia
Primary myelofibrosis
Chronic neutrophilic leukaemia
Chronic eosinophilic leukaemia
Juvenile myelomonocytic leukaemia
Myeloproliferative neoplasm, not otherwise specified

### Chronic myeloid leukaemia risk factors are refined, and accelerated phase is no longer required

Chronic myeloid leukaemia (CML) is defined by the *BCR*::*ABL1* fusion resulting from t(9;22)(q34;q11). The natural history of untreated CML before the introduction of targeted tyrosine kinase inhibitors (TKI) was biphasic or triphasic: an initial indolent CP followed by a blast phase (BP), with or without an intervening accelerated phase (AP). With TKI therapy and careful disease monitoring, the incidence of progression to advanced phase disease has decreased, and the 10-year overall survival rate for CML is 80–90% [10, 11]. The designation of AP has thus become less relevant, where resistance stemming from *ABL1* kinase mutations and/or additional cytogenetic abnormalities and the development of BP represent key disease attributes [12, 13]. Accordingly, AP is omitted in the current classification in favour of an emphasis on high risk features associated with CP progression and resistance to TKI. Criteria for BP include: (1) ≥20% myeloid blasts in the blood or bone marrow; or (2) the presence of an extramedullary proliferation of blasts; or (3) the presence of increased lymphoblasts in peripheral blood or bone marrow. The optimal cutoff for lymphoblasts and the significance of low-level B-lymphoblasts remain unclear and require additional studies.

### Minor changes in diagnostic criteria for *BCR::ABL1*-negative myeloproliferative neoplasms

The classification retains an emphasis on distinguishing between polycythaemia vera (PV), essential thrombocythaemia (ET) and primary myelofibrosis (PMF) using diagnostic criteria established in previous editions, with minor refinements. Distinction between these types is based on integrating peripheral blood findings with molecular data and bone marrow morphologic evaluation findings, as none of these parameters alone provide sufficient diagnostic specificity.

Major diagnostic criteria for the diagnosis of PV include elevated haemoglobin concentration and/or haematocrit, accompanied by trilineage hyperplasia (panmyelosis), with pleomorphic mature megakaryocytes in the bone marrow, and NM_004972:JAK2 p.V617F or *JAK2* exon 12 mutations. As the determination of increased red cell mass with ^51^Cr-labeled red cells has become uncommon in routine clinical practice, it has been removed as a diagnostic criterion. The diagnostic criteria of ET are well-established and have not changed.

Primary myelofibrosis (PMF) is characterized by a proliferation of abnormal megakaryocytes and granulocytes in the bone marrow, which is associated in fibrotic stages with a polyclonal increase in fibroblasts that drive secondary reticulin and/or collagen marrow fibrosis, osteosclerosis, and extramedullary haematopoiesis. Recognizing prefibrotic PMF remains necessary to separate it not only from ET and PV but also from fibrotic PMF [14]. The importance of serial monitoring of bone marrow fibrosis and spleen size using reproducible and standardized criteria remain pertinent, especially for patients receiving JAK1/2 inhibitors. PV and ET progress to AP (10-19% blasts) and BP (≥20% blasts) in a minority of cases, but leukaemic transformation is more frequent in PMF, and leukaemia-free survival is shorter in fibrotic than prefibrotic PMF [15, 16].

While *JAK2*, *CALR*, and *MPL* mutations are considered driver events, mutations in other genes – particularly *TET2*, *ASXL1*, and *DNMT3A –* are found in over half of patients with MPN. Mutations affecting splicing regulators (*SRSF2, SF3B1, U2AF1, ZRSR2*) and other regulators of chromatin structure, epigenetic functions and cellular signaling (e.g., *EZH2, IDH1, IDH2, CBL, KRAS, NRAS, STAG2, TP53*) are less common. These additional mutations are more frequent in PMF and advanced disease compared to PV and ET, and some are known to correlate with a poorer prognostic risk (e.g., *EZH2, IDH1, IDH2, SRSF2, U2AF1*, and *ASXL1* mutations in PMF).

Chronic neutrophilic leukaemia (CNL) is a *BCR*::*ABL1*-negative MPN characterized by sustained peripheral blood neutrophilia (white blood cell count (WBC) ≥ 25 × 10^9^/ L, with ≥80% segmented neutrophils and bands), bone marrow hypercellularity due to neutrophilic granulocyte proliferation, and hepatosplenomegaly. *CSF3R* mutations are common in this disease and detected in >60% of cases [17, 18].

Chronic eosinophilic leukaemia (CEL) is a multi-system disorder characterized by a sustained *clonal* proliferation of morphologically abnormal eosinophils and eosinophil precursors resulting in persistent hypereosinophilia in blood and bone marrow [19–21]. Several changes to the diagnostic criteria of CEL are introduced: (1) the time interval required to define sustained hypereosinophilia is reduced from 6 months to 4 weeks; (2) addition of requirement for both clonality and abnormal bone marrow morphology (e.g., megakaryocytic or erythroid dysplasia); and, (3) elimination of increased blasts (≥2% in peripheral blood or 5-19% in bone marrow) as an alternative to clonality. These criteria improve the distinction between CEL and entities such as idiopathic hypereosinophilic syndrome and hypereosinophilia of unknown significance [22]. As the criteria of CEL and its place relative to other disorders with eosinophilia have become well characterized, the qualifier “not otherwise specified” is no longer needed and has been omitted from the name.

As in prior editions, MPN, not otherwise specified (MPN-NOS) is a designation that should be reserved for cases with clinical, laboratory, morphologic, and molecular features of MPN but lacking diagnostic criteria of any specific MPN type or with features that overlap across distinct MPN types.

### Juvenile myelomonocytic leukaemia is recognized as a myeloproliferative neoplasm of early childhood with frequent association with germline pathogenic gene variants

Juvenile myelomonocytic leukaemia (JMML) is a haematopoietic stem cell-derived myeloproliferative neoplasm of early childhood. The pathogenetic mechanism in at least 90% of cases involves unchecked activation of the RAS pathway. A diagnosis of JMML can be made by combining clinical, laboratory, and molecular criteria. Updates to diagnostic criteria include: (1) exclusion of *KMT2A* rearrangements; (2) elimination of monosomy 7 as a cytogenetic criterion; and, (3) emphasizing the significance of diagnostic molecular studies, particularly those aimed at demonstrating RAS pathway activation. The genetic background of JMML plays a major role in risk stratification and therapeutic approaches, with cases initiated by somatic mutations involving *PTPN11* and germline pathogenic variants associated with neurofibromatosis type 1 being the most aggressive types, while some cases associated with pathogenic germline *CBL* variants undergoing occasionally spontaneous remission. The inclusion of JMML under MPN reflects its molecular pathogenesis and underscores the virtual absence of stigmata of bona fide myelodysplastic neoplasia in this disease.

#### Summary Box:

- CML phases consolidated into chronic and blast phases, with emphasis on risk features in chronic phase.
- Diagnostic criteria of CEL are updated, and the qualifier NOS is omitted.
- JMML is categorized under myeloproliferative neoplasms.

## Mastocytosis

Mastocytosis comprises rare heterogeneous neoplasms characterized by an accumulation of abnormal mast cells in various organs or tissues, typically driven by constitutive activation of the KIT receptor. The pathology of mastocytosis is complex, and clinical features span a broad spectrum that may be modulated by the presence of comorbidities. Significant comorbidities include IgE-dependent allergies, vitamin D deficiency, and psychiatric, psychological or mental problems. The classification continues to recognize three disease types: systemic mastocytosis (SM), cutaneous mastocytosis (CM) and mast cell sarcoma (MCS) [23]. (Table 2)

**Table 2:** Mastocytosis types and subtypes.

**Cutaneous mastocytosis**
Urticaria pigmentosa/Maculopapular cutaneous mastocytosis
Monomorphic
Polymorphic
Diffuse cutaneous mastocytosis
Cutaneous mastocytoma
Isolated mastocytoma
Multilocalized mastocytoma
**Systemic mastocytosis**
Bone marrow mastocytosis
Indolent systemic mastocytosis
Smoldering systemic mastocytosis
Aggressive systemic mastocytosis
Systemic mastocytosis with an associated haematologic neoplasm
Mast cell leukemia
**Mast cell sarcoma**

Note: Well-differentiated systemic mastocytosis (WDSM) represents a morphologic variant that may occur in any SM type/subtype, including mast cell leukaemia.

A somatic point mutation in the *KIT* gene at codon 816 is detected in >90% of patients with SM. Other rare activating *KIT* alterations include mutations in the extracellular (e.g., deletion of codon 419 on exon 8 or A502_Y503dup in exon 9), transmembrane (e.g., NM_000222:KIT p.F522C), or juxtamembrane (e.g., NM_000222:KIT p.V560G) domains, detected in <1% of advanced SM cases but enriched in cases of indolent SM. Most patients with advanced SM and NM_000222:*KIT* p.D816V have additional somatic mutations involving most frequently *TET2, SRSF2, ASXL1, RUNX1*, and *JAK2*. An associated haematologic (usually myeloid) neoplasm may be detected in these patients [24].

Diagnostic criteria for SM have been modified. Namely, expression of CD30 and the presence of any *KIT* mutation causing ligand-independent activation have been accepted as minor diagnostic criteria. Basal serum tryptase level >20 ng/ml, which should be adjusted in case of hereditary alpha-tryptasaemia, is a minor SM criterion [25]. In addition, bone marrow mastocytosis is now a separate subtype of SM characterized by absence of skin lesions and B-findings and a basal serum tryptase below 125 ng/ml. Classical B-findings (‘burden of disease’) and C-findings (‘cytoreduction-requiring’) have undergone minor refinements. Most notably, NM_000222:*KIT* p.D816V mutation with VAF ≥ 10% in bone marrow cells or peripheral blood leukocytes qualifies as a B-finding.

The classification recognizes well-differentiated systemic mastocytosis (WDSM) as a morphologic pattern that can occur in any SM subtype, characterized by round and well-granulated mast cells usually heavily infiltrating the bone marrow. In most patients with WDSM, *KIT* codon 816 mutation is not detected, and neoplastic mast cells are usually negative for CD25 and CD2 but positive for CD30 [26].

### Summary Box:

- Diagnostic criteria for mastocytosis have been refined: CD30 and any *KIT* mutation are introduced as minor diagnostic criteria.
- Bone marrow mastocytosis is a new SM subtype.
- *KIT* D816V mutation with VAF ≥ 10% qualifies as a B-finding.

## Myelodysplastic neoplasms

### New terminology and grouping framework

The classification introduces the term *myelodysplastic neoplasms* (abbreviated MDS) to replace myelodysplastic syndromes, underscoring their neoplastic nature and harmonizing terminology with MPN. These clonal haematopoietic neoplasms are defined by cytopenias and morphologic dysplasia. As indicated above, cytopenia definitions are adopted for consistency across CCUS, MDS, and MDS/MPN. Additionally, the recommended threshold for dysplasia is set at 10% for all lineages. MDS entities are now grouped as those having *defining genetic abnormalities* and those that are *morphologically defined*. (Table 3) It is posited that such reorganization enhances classification rigor by emphasizing genetically-defined disease types and ceding the prior emphasis on ‘risk-based’ grouping in the classification (based on blast percentage, ring sideroblasts, and number of lineages with dysplasia) in favour of more comprehensive risk-stratification schemes such as the Revised International Prognostic Scoring System for MDS (IPSS-R) [27]. An additional modification is a clarified terminology to distinguish between MDS with *low* blasts (MDS-LB) and MDS with *increased* blasts (MDS-IB), while retaining longstanding cutoffs.

**Table 3:** Classification and defining features of myelodysplastic neoplasms (MDS).

	Blasts	Cytogenetics	Mutations
**MDS with defining genetic abnormalities**			
MDS with low blasts and isolated 5q deletion (MDS-5q)	<5% BM and <2% PB	5q deletion alone, or with 1 other abnormality other than monosomy 7 or 7q deletion	
MDS with low blasts and *SF3B1* mutation^a^ (MDS-*SF3B1*)		Absence of 5q deletion, monosomy 7, or complex karyotype	*SF3B1*
MDS with biallelic *TP53* inactivation (MDS-bi*TP53*)	<20% BM and PB	Usually complex	Two or more *TP53* mutations, or 1 mutation with evidence of *TP53* copy number loss or cnLOH
**MDS, morphologically defined**			
MDS with low blasts (MDS-LB)	<5% BM and <2% PB		
MDS, hypoplastic^b^ (MDS-h)			
MDS with increased blasts (MDS-IB)			
MDS-IB1	5–9% BM or 2–4% PB		
MDS-IB2	10-19% BM or 5–19% PB or Auer rods		
MDS with fibrosis (MDS-f)	5–19% BM; 2–19% PB		

^a^Detection of ≥15% ring sideroblasts may substitute for *SF3B1* mutation. Acceptable related terminology: MDS with low blasts and ring sideroblasts.

^b^By definition, ≤25% bone marrow cellularity, age adjusted.

*BM* bone marrow, *PB* peripheral blood, *cnLOH* copy neutral loss of heterozygosity.

### MDS with defining genetic abnormalities

Myelodysplastic neoplasms with defining genetic abnormalities are grouped together and include: *MDS with low blasts and isolated 5q deletion* (MDS-5q), *MDS with low blasts and SF3B1 mutation* (MDS-*SF3B1*), and *MDS with biallelic TP53 inactivation* (MDS-bi*TP53*). The latter supersedes MDS-5q and MDS-*SF3B1*.

The diagnostic criteria of MDS-5q have not changed. While recognized as factors that may potentially alter the biology and/or prognosis of the disease, the presence of *SF3B1* or a *TP53* mutation (not multi-hit) does not per se override the diagnosis of MDS-5q.

Recent studies have identified MDS-*SF3B1* as a distinct disease type that includes over 90% of MDS with ≥5% ring sideroblasts [28]. The term *MDS with low blasts and ring sideroblasts* is retained as an acceptable alternative to be used for cases with wild-type *SF3B1* and ≥15% ring sideroblasts. This permits inclusion of rare MDS cases harbouring driver mutations in other RNA splicing components.

Pathogenic *TP53* alterations of any type (sequence variations, segmental deletions and copy neutral loss of heterozygosity) are detected in 7-11% of MDS [29–31]. Among these, about two-thirds of patients have multiple *TP53* hits (multi-hit), consistent with biallelic *TP53* alterations [29]. Biallelic *TP53* (bi*TP53*) alterations may consist of multiple mutations or mutation with concurrent deletion of the other allele. This “multi-hit” mutational status results in a neoplastic clone that lacks any residual wild-type p53 protein. Clinical detection of biallelic *TP53* alterations is based on sequencing analysis (covering at least exons 4 to 11) [29, 32], often coupled with a technique to detect copy number status, usually fluorescence in situ hybridization with a probe set specific for the *TP53* locus on 17p13.1 and/or array techniques (e.g., comparative genomic hybridization or single nucleotide polymorphism arrays) [33]. Loss of genetic material at the *TP53* locus may also be inferred by next-generation sequencing [29]. A *TP53* VAF ≥ 50% may be regarded as presumptive (not definitive) evidence of copy loss on the trans allele or copy neutral loss of heterozygosity when a constitutional *TP53* variant can be ruled out. When two or more *TP53* mutations are detected, they usually affect both alleles [29] and can be considered a multi-hit status. Over 90% of patients with MDS-bi*TP53* have complex, mostly very complex (>3), karyotype [29, 30] and thus are regarded as very high risk in IPSS-R [27]. Additional studies are needed to determine whether bi*TP53* status is per se AML-defining, a point for consideration in future editions. Notwithstanding, published data suggests that MDS-bi*TP53* may be regarded as AML-equivalent for therapeutic considerations [29, 30].

### MDS, morphologically defined

Hypoplastic MDS (MDS-h) is listed as a distinct MDS type in this edition. Long recognized as having distinctive features, MDS-h is associated with a T-cell mediated immune attack on haematopoietic stem and progenitor cells, along with oligoclonal expansion of CD8 + cytotoxic T-cells overproducing IFNγ and/or TNFα. Several features overlap across the triad of MDS-h, paroxysmal nocturnal haemoglobinuria (PNH) and aplastic anaemia (AA), including an association with CH [34–36]. Many patients with MDS-h have sustainable responses to agents used in patients with AA (i.e., anti-thymocyte globulin, ATG). As such, an emphasis is placed on careful morphologic evaluation, typically requiring trephine biopsy evaluation in addition to evaluation of bone marrow smears and touch preparations, and detection of mutations and/or clonal cytogenetic abnormalities. Individuals with germline pathogenic variants in *GATA2*, *DDX41*, Fanconi anaemia (FA) or telomerase complex genes can have hypoplastic bone marrow and evolve to MDS and/or AML and do not respond to immunosuppressive treatment.

As the number of dysplastic lineages is usually dynamic and often represents clinical and phenotypic manifestation of clonal evolution – rather than per se defining a specific MDS type, the distinction between single lineage and multilineage dysplasia is now considered optional. The updated MDS classification scheme and the incorporation of CCUS in the classification obviates the need for “NOS” or “unclassifiable” attributes. Specifically, MDS, unclassifiable, which was present in the prior edition, is removed.

### The boundary between MDS and AML is softened, but the 20% blast cutoff to define AML is retained

Reassessment of the bone marrow blast percentage defining the boundary of MDS-IB2 and AML has been advocated for several cogent reasons and in view of novel therapeutic approaches that show efficacy in patients currently classified as MDS or AML with 10-30% myeloid blasts [37–39]. Salient practical challenges underpinning arguments for such a reassessment include: (1) any blast-based cutoff is arbitrary and cannot reflect the biologic continuity naturally inherent in myeloid pathogenic mechanisms; (2) blast enumeration is subject to sampling variations/error and subjective evaluation; and, (3) no gold standard for blast enumeration exists, and orthogonal testing platforms can and often do produce discordant results. The pros and cons of merging MDS-IB2 with AML and adopting a 10% cutoff for what would be called MDS/AML were explored in multidisciplinary expert discussions and at editorial board meetings in the course of producing this classification. Lowering the blast cutoff to define AML was felt to suffer from the same challenges listed above and would merely replace one cutoff with another. Further, an arbitrary cutoff of 10% blasts to define AML (even if qualified as MDS/AML or AML/MDS) carries a risk of overtreatment. Accordingly, a balanced approach was adopted by eliminating blast cutoffs for most AML types with defining genetic alterations but retaining a 20% blast cutoff to delineate MDS from AML. Notwithstanding, there was broad agreement that MDS-IB2 may be regarded as AML-equivalent for therapeutic considerations and from a clinical trial design perspective when appropriate.

### Childhood myelodysplastic neoplasms: Enhanced specificity of disease terminology introduced

Childhood MDS is a clonal haematopoietic stem cell neoplasm arising in children and adolescents (<18 years of age) leading to ineffective haematopoiesis, cytopenia(s), and risk of progression to AML. The annual incidence is 1-2 per million children, with 10-25% presenting with increased blasts. JMML, myeloid proliferations associated with Down syndrome, and MDS post cytotoxic therapy are excluded from this group and belong elsewhere in the classification. The qualifying term *childhood* MDS emphasizes that this category of myeloid neoplasms is biologically distinct from that seen in adults [40, 41], underscoring the need to further elucidate its pathogenesis which remains incompletely understood

*Childhood MDS with low blasts* (cMDS-LB) replaces the former term “refractory cytopenia of childhood (RCC)”. It includes two subtypes: childhood MDS with low blasts, hypocellular; and, childhood MDS with low blasts, not otherwise specified (NOS). (Table 4) Exclusion of non-neoplastic causes of cytopenia such as infections, nutritional deficiencies, metabolic diseases, bone marrow failure syndromes (BMFS), and germline pathogenic variants remains an essential diagnostic prerequisite for childhood MDS with low blasts. Approximately 80% of cases show hypocellular bone marrow with features similar to severe aplastic anemia and other BMFS, requiring close morphologic examination to evaluate the distribution, maturation, and presence of dysplasia in haematopoietic lineages [42]. Some cytogenetic findings such as monosomy 7, 7q deletion, or complex karyotype are associated with an increased risk of progression to AML and typically treated with haematopoietic stem cell transplantation, while cases with normal karyotype or trisomy 8 can have an indolent course.

**Table 4:** Childhood myelodysplastic neoplasms (MDS).

	Blasts
**Childhood MDS with low blasts**	<5% BM; <2% PB
Hypocellular	
Not otherwise specified	
**Childhood MDS with increased blasts**	5–19% BM; 2–19% PB

*BM* bone marrow, *PB* peripheral blood.

*Childhood MDS with increased blasts* (cMDS-IB) is defined as having ≥5% blasts in the bone marrow or ≥2% blasts in the peripheral blood. The genetic landscape of cMDS-IB and cMDS-LB is similar, and they both differ from MDS arising in adults. Acquired cytogenetic abnormalities and RAS-pathway mutations are more common in cMDS-IB compared to cMDS-LB [43, 44].

#### Summary Box:

- Myelodysplastic syndromes renamed myelodysplastic neoplasms (abbreviated MDS).
- MDS genetic types updated to include MDS-5q, MDS-*SF3B1* and MDS-bi*TP53*
- Hypoplastic MDS (MDS-h) is recognized as a distinct disease type.
- MDS with low blasts (MDS-LB) is a new term that enhances clarity.
- MDS with increased blasts (MDS-IB) is a new term that enhances clarity.
- Terminology of childhood MDS types is updated.

## Myelodysplastic/myeloproliferative neoplasms

This category of myeloid neoplasms is defined by overlapping pathologic and molecular features of MDS and MPN, often manifesting clinically with various combinations of cytopenias and cytoses. The definition of cytopenias is the same as that for MDS. The classification includes major revisions in the diagnostic criteria of CMML and terminology changes for other MDS/MPN types. (Table 5)

**Table 5:** Myelodysplastic/myeloproliferative neoplasms.

Chronic myelomonocytic leukaemia
Myelodysplastic/myeloproliferative neoplasm with neutrophilia
Myelodysplastic/myeloproliferative neoplasm with *SF3B1* mutation and thrombocytosis
Myelodysplastic/myeloproliferative neoplasm, not otherwise specified

### Chronic myelomonocytic leukaemia diagnostic criteria, subtypes, and blast-based subgrouping criteria reflect diagnostic refinement and emphasize unifying characteristics

The prototype and most common MDS/MPN is chronic myelomonocytic leukaemia (CMML), which is characterized by sustained peripheral blood monocytosis and various combinations of somatic mutations involving epigenetic regulation, spliceosome, and signal transduction genes. Diagnostic criteria are revised to include prerequisite and supporting criteria. (Table 6) The first prerequisite criterion is persistent absolute (≥0.5 × 10^9^/ L) and relative (≥10%) peripheral blood monocytosis. Namely, the cutoff for absolute monocytosis is lowered from 1.0 ×10^9^/L to 0.5 ×10^9^/L to incorporate cases formerly referred to as oligomonocytic CMML [45–47]. To enhance diagnostic accuracy when absolute monocytosis is ≥0.5 ×10^9^/L but <1.0 ×10^9^/L, detection of one of more clonal cytogenetic or molecular abnormality and documentation of dysplasia in at least one lineage are required. Abnormal partitioning of peripheral blood monocyte subsets is introduced as a new supporting criterion [48, 49]. Additional studies are needed to determine the optimal approach to classifying individuals with unexplained clonal monocytosis [50] who do not fit the new diagnostic criteria of CMML.

**Table 6:** Diagnostic criteria of chronic myelomonocytic leukaemia.

**Prerequisite criteria**
1. Persistent absolute (≥0.5 × 10^9^/ L) and relative (≥10%) peripheral blood monocytosis.
2. Blasts constitute <20% of the cells in the peripheral blood and bone marrow.^a^
3. Not meeting diagnostic criteria of chronic myeloid leukaemia or other myeloproliferative neoplasms.^b^
4. Not meeting diagnostic criteria of myeloid/lymphoid neoplasms with tyrosine kinase fusions.^c^
**Supporting criteria**
1. Dysplasia involving ≥1 myeloid lineages.^d^
2. Acquired clonal cytogenetic or molecular abnormality.
3. Abnormal partitioning of peripheral blood monocyte subsets.^e^
**Requirements for diagnosis**
- Pre-requisite criteria must be present in all cases.
- If monocytosis is ≥ 1 × 10^9^/ L: one or more supporting criteria must be met.
- If monocytosis is ≥0.5 and <1 × 10^9^/ L: supporting criteria 1 and 2 must be met.
**Subtyping criteria**
- Myelodysplastic CMML (MD-CMML): WBC < 13 × 10^9^/L
- Myeloproliferative CMML (MP-CMML): WBC ≥ 13 × 10^9^/L
**Subgrouping criteria** (based on percentage of blasts and promonocytes)
CMML-1: <5% in peripheral blood and <10% in bone marrow
CMML-2: 5–19% in peripheral blood and 10-19% in bone marrow

^a^Blasts and blast equivalents include myeloblasts, monoblasts and promonocytes.

^b^Myeloproliferative neoplasms (MPN) can be associated with monocytosis at presentation or during the course of the disease; such cases can mimic CMML. In these instances, a documented history of MPN excludes CMML. The presence of MPN features in the bone marrow and/or high burden of MPN-associated mutations (*JAK2, CALR* or *MPL*) tends to support MPN with monocytosis rather than CMML.

^c^Criteria for myeloid/lymphoid neoplasms with tyrosine kinase fusions should be specifically excluded in cases with eosinophilia.

^d^Morphologic dysplasia should be present in ≥10% of cells of a haematopoietic lineage in the bone marrow.

^e^Based on detection of increased classical monocytes (>94%) in the absence of known active autoimmune diseases and/or systemic inflammatory syndromes.

Two disease subtypes with salient clinical and genetic features are now formally recognized based on WBC: *myelodysplastic* CMML (MD-CMML) (WBC < 13 × 10^9^/L) and *myeloproliferative* CMML (MP-CMML) (WBC ≥ 13 × 10^9^/L). MP-CMML is commonly associated with activating RAS pathway mutations and adverse clinical outcomes [51]. The blast-based subgroup of CMML-0 (<2% blasts in blood and <5% blasts in bone marrow) introduced in the previous edition has been eliminated in view of evidence that its addition provides no or limited prognostic significance [52, 53].

### Atypical chronic myeloid leukaemia is renamed MDS/MPN with neutrophilia, and other terminology updates

Diagnostic criteria for other MDS/MPN types were largely unchanged. The term *MDS/MPN with neutrophilia* replaces the term atypical CML. This change underscores the MDS/MPN nature of the disease and avoids potential confusion with CML. MDS/MPN with ring sideroblasts and thrombocytosis is redefined based on *SF3B1* mutation and renamed *MDS/MPN with SF3B1 mutation and thrombocytosis*. The term MDS/MPN with ring sideroblasts and thrombocytosis has been retained as an acceptable term to be used for cases with wild-type *SF3B1* and ≥15% ring sideroblasts. MDS/MPN, unclassifiable is now termed *MDS/MPN, not otherwise specified*; this is in line with an intentional effort to remove the paradoxical qualifier “unclassifiable” from the entire classification.

#### Summary Box:

- CMML diagnostic criteria undergo major revisions, including lowering the cutoff for absolute monocytosis, adopting MD-CMML and MP-CMML subtypes, and eliminating CMML-0.
- Atypical chronic myeloid leukaemia renamed MDS/MPN with neutrophilia.
- MDS/MPN with ring sideroblasts and thrombocytosis redefined based on *SF3B1* mutation and renamed MDS/MPN with *SF3B1* mutation and thrombocytosis.

## Acute myeloid leukaemia

### Enhanced grouping framework permitting scalable genetic classification and deemphasizing blast enumeration where relevant

The classification of AML is re-envisioned to emphasize major breakthroughs over the past few years in how this disease is understood and managed. Foremost is the separation of AML with defining genetic abnormalities from AML defined by differentiation. (Table 7) The latter eliminates the previously confusing use of the term AML NOS, under which types based on differentiation were listed. Another key change, as indicated above, is the elimination of the 20% blast requirement for AML types with defining genetic abnormalities (with the exception of AML with *BCR*::*ABL1* fusion and AML with *CEBPA* mutation). Removal of the blast cutoff requires correlation between morphologic findings and the molecular genetic studies to ensure that the defining abnormality is driving the disease pathology. This approach was deemed more appropriate than assigning another arbitrary lower bone marrow blast cutoff. A third component of the new structure is the introduction of a section on AML with *other defined genetic alterations*, a landing spot for new and/or uncommon AML subtypes that may (or may not) become defined types in future editions of the classification. As such, the overall AML classification structure continues to emphasize integration of clinical, molecular/genetic, and pathologic parameters and emphasis on clinicopathologic judgement.

**Table 7:** Acute myeloid leukaemia.

**Acute myeloid leukaemia with defining genetic abnormalities**
Acute promyelocytic leukaemia with *PML*::*RARA* fusion
Acute myeloid leukaemia with *RUNX1*::*RUNX1T1* fusion
Acute myeloid leukaemia with *CBFB*::*MYH11* fusion
Acute myeloid leukaemia with *DEK*::*NUP214* fusion
Acute myeloid leukaemia with *RBM15*::*MRTFA* fusion
Acute myeloid leukaemia with *BCR*::*ABL1* fusion
Acute myeloid leukaemia with *KMT2A* rearrangement
Acute myeloid leukaemia with *MECOM* rearrangement
Acute myeloid leukaemia with *NUP98* rearrangement
Acute myeloid leukaemia with *NPM1* mutation
Acute myeloid leukaemia with *CEBPA* mutation
Acute myeloid leukaemia, myelodysplasia-related
Acute myeloid leukaemia with other defined genetic alterations
**Acute myeloid leukaemia, defined by differentiation**
Acute myeloid leukaemia with minimal differentiation
Acute myeloid leukaemia without maturation
Acute myeloid leukaemia with maturation
Acute basophilic leukaemia
Acute myelomonocytic leukaemia
Acute monocytic leukaemia
Acute erythroid leukaemia
Acute megakaryoblastic leukaemia

### AML with defining genetic abnormalities

While the classification retains much of the established diagnostic criteria for AML with *PML*::*RARA*, AML with *RUNX1*::*RUNX1T1*, and AML with *CBF*::*MYH11*, increased recognition of the importance of highly sensitive measurable residual disease (MRD) evaluation techniques, and the impact of concurrent molecular alterations reflect factors that impact patient management and therapeutic decisions in current practice. Namely, prognostic factors have expanded from *KIT* mutations, which are still relevant, to include additional cytogenetic features and MRD status post induction. The diagnostic criteria of AML with *DEK*::*NUP214* and AML with *RBM15*::*MRTFA* (formerly *RBM15*::*MKL1*) have also remained largely unchanged.

AML with *BCR*::*ABL1* and AML with *CEBPA* mutation are the only disease types with a defined genetic abnormality that require at least 20% blasts for diagnosis. The blast cutoff requirement is needed for the former to avoid overlap with CML. Distinguishing AML with *BCR*::*ABL1* from initial myeloid blast phase of CML can be challenging, and additional evidence continues to be needed to better characterize this AML type. There is insufficient data to support any change in the blast cutoff criterion for AML with *CEBPA* mutation [54, 55].

Three AML types with characteristic rearrangements involving *KMT2A, MECOM*, and *NUP98* are recognized. A blast count under 20% is acceptable based on studies demonstrating that patients with <20% blasts (MDS) and any of these rearrangements have clinical features that resemble those with higher blast counts. It is important to note that rearrangements involving these three genes, particularly *NUP98*, may be cryptic on conventional karyotyping. *AML with KMT2A rearrangement* is the new term that replaces “AML with t(9;11)(p22;q23); *KMT2A-MLLT3”*. More than 80 *KMT2A* fusion partners have been described, with *MLLT3, AFDN, ELL*, and *MLLT10* being most common. While not required, the identification of the fusion partner is desirable since it could provide prognostic information and may impact disease monitoring. Adult patients often present with high blast counts, usually with monocytic differentiation. In children particularly, AML with *KMT2A*::*MLLT3* and *KMT2A*::*MLLT10* show megakaryoblastic differentiation and/or low blast counts in bone marrow aspirate smears.

AML defined by mutations include AML with *NPM1* and AML with *CEBPA* mutation. AML with *NPM1* mutation can be diagnosed irrespective of the blast count, albeit again with emphasis on judicious clinicopathologic correlation. This approach aligns with data showing that cases previously classified as MDS or MDS/MPN with *NPM1* progress to AML in a short period of time. Similar data have emerged from patients with CH who acquire *NPM1* mutation. The definition of AML with *CEBPA* mutation has changed to include biallelic (biCEBPA) as well as single mutations located in the basic leucine zipper (bZIP) region of the gene (smbZIP-*CEBPA*). The favourable prognosis associated with smbZIP-*CEBPA* has been demonstrated in cohorts of children and adults up to 70 years old. *RUNX1* mutations in AML overlap with such a broad range of defining molecular features that it was determined to lack enough specificity to define a standalone AML type.

Several changes were introduced to the entity formerly designated AML with myelodysplasia-related changes, now called *AML, myelodysplasia-related* (AML-MR). This AML type is defined as a neoplasm with ≥20% blasts expressing a myeloid immunophenotype and harboring specific cytogenetic and molecular abnormalities associated with MDS, arising *de novo* or following a known history of MDS or MDS/MPN. Key changes include: (1) removal of morphology alone as a diagnostic premise to make a diagnosis of AML-MR; (2) update of defining cytogenetic criteria; and, (3) introduction of a mutation-based definition based on a set of 8 genes – *SRSF2, SF3B1, U2AF1, ZRSR2, ASXL1, EZH2, BCOR, STAG2*, > 95% of which are present specifically in AML arising post MDS or MDS/MPN [56, 57]. The presence of one or more cytogenetic or molecular abnormalities listed in Table 8 and/or history of MDS or MDS/MPN are required for diagnosing AML-MR.

**Table 8:** Cytogenetic and molecular abnormalities defining acute myeloid leukaemia, myelodysplasia-related.

Defining cytogenetic abnormalities
Complex karyotype (≥3 abnormalities)
5q deletion or loss of 5q due to unbalanced translocation
Monosomy 7, 7q deletion, or loss of 7q due to unbalanced translocation
11q deletion
12p deletion or loss of 12p due to unbalanced translocation
Monosomy 13 or 13q deletion
17p deletion or loss of 17p due to unbalanced translocation
Isochromosome 17q
idic(X)(q13)
**Defining somatic mutations**
*ASXL1*
*BCOR*
*EZH2*
S*F3B1*
*SRSF2*
*STAG2*
*U2AF1*
*ZRSR2*

AML with *other defined genetic alterations* represents a landing spot for new, often rare, emerging entities whose recognition is desirable to determine whether they might constitute distinct types in future editions. At present, subtypes under this heading include AML with rare genetic fusions.

### AML defined by differentiation

This AML family includes cases that lack defining genetic abnormalities. (Table 9) It is anticipated that the number of such cases will diminish as discoveries provide novel genetic contexts for their classification. Notwithstanding, categorizing AML cases lacking defining genetic abnormalities based on differentiation offers a longstanding classification paradigm with practical, prognostic, and perhaps therapeutic implications.

**Table 9:** Differentiation markers and criteria for acute myeloid leukaemia (AML) types defined by differentiation.

Type	Diagnostic criteria*
AML with minimal differentiation	• Blasts are negative (<3%) for MPO and SBB by cytochemistry
	• Expression of two or more myeloid-associated antigens, such as CD13, CD33, and CD117
AML without maturation	• ≥3% blasts positive for MPO (by immunophenotyping or cytochemistry) or SBB and negative for NSE by cytochemistry
	• Maturing cells of the granulocytic lineage constitute <10% of the nucleated bone marrow cells
	• Expression of two or more myeloid-associated antigens, such as MPO, CD13, CD33, and CD117
AML with maturation	• ≥3% blasts positive for MPO (by immunophenotyping or cytochemistry) or SBB by cytochemistry
	• Maturing cells of the granulocytic lineage constitute ≥10% of the nucleated bone marrow cells
	• Monocyte lineage cells constitute < 20% of bone marrow cells
	• Expression of two or more myeloid-associated antigens, such as MPO, CD13, CD33, and CD117
Acute basophilic leukemia	• Blasts & immature/mature basophils with metachromasia on toluidine blue staining
	• Blasts are negative for cytochemical MPO, SBB, and NSE
	• No expression of strong CD117 equivalent (to exclude mast cell leukemia)
Acute myelomonocytic leukaemia	• ≥20% monocytes and their precursors
	• ≥20% maturing granulocytic cells
	• ≥3% of blasts positive for MPO (by immunophenotyping or cytochemistry)
Acute monocytic leukaemia	• ≥80% monocytes and/or their precursors (monoblasts and/or promonocytes)
	• <20% maturing granulocytic cells
	• Blasts and promonocytes expressing at least two monocytic markers including CD11c, CD14, CD36 and CD64, or NSE positivity on cytochemistry
Acute erythroid leukaemia	• ≥30% immature erythroid cells (proerythroblasts)
	• Bone marrow with erythroid predominance, usually ≥80% of cellularity
Acute megakaryoblastic leukaemia	• Blasts express at least one or more of the platelet glycoproteins: CD41 (glycoprotein llb), CD61 (glycoprotein IIIa), or CD42b (glycoprotein lb)^b^

^*^Shared diagnostic criteria include:

-  ≥20% blasts in bone marrow and/or blood (except for acute erythroid leukaemia).

-  Criteria for AML types with defined genetic alterations are not met.

-  Criteria for mixed-phenotype acute leukaemia are not met (relevant for AML with minimal differentiation).

-  Not fulfilling diagnostic criteria for myeloid neoplasm post cytotoxic therapy.

-  No prior history of myeloproliferative neoplasm.

*BM* bone marrow, *MPO* myeloperoxidase, *NSE* nonspecific esterase, *PB* peripheral blood, *SBB* Sudan Black B.

The classification includes an updated comprehensive framework of differentiation markers and criteria, harmonized with those of mixed-phenotype acute leukaemia (MPAL) and early T-precursor lymphoblastic leukaemia/lymphoma (ETP-ALL) (see section below on acute leukaemia of ambiguous lineage). Indeed, the recent identification of *BCL11B* rearrangements in MPAL T/Myeloid, ETP-ALL, acute leukaemia of ambiguous lineage (ALAL) and a subset of AML with minimal differentiation suggests a biologic continuum across these entities, a finding with likely implications on future editions of the classification [58–61].

Acute erythroid leukaemia (AEL) (previously pure erythroid leukaemia, an acceptable related term in this edition) is a distinct AML type characterized by neoplastic proliferation of erythroid cells with features of maturation arrest and high prevalence of biallelic *TP53* alterations. Diagnostic criteria include erythroid predominance, usually ≥80% of bone marrow elements, of which ≥30% are proerythroblasts (or pronormoblasts). The occurrence of AEL cases in which nucleated erythroid cells constitute less than 80% of bone marrow cellularity is recognized; such cases share the same clinicopathologic features of other AEL [62, 63]. The central role that biallelic *TP53* mutations play in this aggressive AML type is underscored [64, 65]. The diagnosis of AEL supersedes AML-MR. *De novo* AEL and cases that arise following MDS or MDS/MPN share distinctive morphologic features, with prominent proerythroblast proliferation. Proerythroblast have been shown to play an important role in treatment resistance and poor prognosis in AML patients [66, 67].

Several molecular drivers can give rise to acute megakaryoblastic leukaemia (AMKL), which arises within three clinical groups: children with Down syndrome, children without Down syndrome, and adults. Immunophenotyping and detection of markers of megakaryocytic differentiation are required to make a diagnosis of AMKL and detect the newly described “RAM immunophenotype”, which correlates with *CBFA2T3*::*GLIS2*, a subtype of *AML with other defined genetic alterations*.

### Myeloid sarcoma

Myeloid sarcoma represents a unique tissue-based manifestation of AML or transformed MDS, MDS/MPN, or MPN. Cases of *de novo* myeloid sarcoma should be investigated comprehensively, including cytogenetic and molecular studies, for appropriate classification and planning therapy. Molecular alterations in myeloid sarcoma and concurrent bone marrow disease are concordant in ~70% of patients, suggesting that myeloid sarcoma may be derived from a common haematopoietic stem cell or precursor [68, 69]. Relevant gene mutations are detected in a subset of patients with morphologically normal-appearing bone marrow, suggesting low-level clonal myeloid disease or CH in the bone marrow [68, 70].

#### Summary Box:

- AML is arranged into two families: AML with *defining genetic abnormalities* and AML *defined by differentiation*. AML, NOS is no longer applicable.
- Most AML with defining genetic abnormalities may be diagnosed with <20% blasts.
- AML-MR replaces the former term AML “with myelodysplasia-related changes”, and its diagnostic criteria are updated. AML transformation of MDS and MDS/MPN continues to be defined under AML-MR in view of the broader unifying biologic features.
- AML with rare fusions are incorporated as subtypes under AML with *other defined genetic alterations*.
- AML with somatic *RUNX1* mutation is not recognized as a distinct disease type due to lack of sufficient unifying characteristics.

## Secondary myeloid neoplasms

### A newly segregated category encompassing diseases that arise in the setting of certain known predisposing factors

Myeloid neoplasms that arise secondary to exposure to cytotoxic therapy or germline predisposition are grouped in this category. AML transformation of MPN is retained in the MPN category, while AML transformation of MDS and MDS/MPN is kept under AML-MR (see above). The framework of this disease category was redesigned with an eye towards two important areas: (1) providing a scalable structure for incorporating novel discoveries in the area of germline predisposition to myeloid neoplasia; (2) recognizing the dual importance of cataloguing myeloid neoplasms that arise following exposure to cytotoxic therapies for clinical purposes as well as population health purposes. The latter factor is gaining increased recognition as cancer survival is prolonged and the incidence of late complications of therapy such as secondary myeloid neoplasia increases. An overarching principle in this context is the requirement to consider “post cytotoxic therapy” and “associated with germline [gene] variant” as disease attributes that should be added as qualifiers to relevant myeloid disease types whose criteria are fulfilled as defined elsewhere in the classification, e.g. *AML with KMT2A rearrangement post cytotoxic therapy* or *MDS with low blasts associated with germline RUNX1 variant*.

### Myeloid neoplasms post cytotoxic therapy: introduction of more precise terminology and novel associations with new cytotoxic drug classes

As in previous editions, this category includes AML, MDS, and MDS/MPN arising in patients exposed to cytotoxic (DNA-damaging) therapy for an unrelated condition. The terminology and definitions of this disease category have been modified slightly to reflect an improved understanding of the risk that CH plays as a risk factor for myeloid neoplasia related particularly to the expansion of pre-existing clones secondary to selection pressures of cytotoxic therapy agents in an altered marrow environment [71]. Thus, the diagnosis of myeloid neoplasms post cytotoxic therapy (MN-pCT) entails fulfilment of criteria for a myeloid neoplasm in addition to a documented history of chemotherapy treatment or large-field radiation therapy for an unrelated neoplasm [72]. This would exclude CCUS, which by definition lacks sufficient support for morphologic dysplasia. Cases with a ‘*de novo* molecular signature’ such as *NPM1* mutation and core-binding factor leukaemias should still be assigned to this category since the “*post cytotoxic therapy*” designation is based on the medical history, and the indication of the most specific diagnosis in the pathology report is recommended when possible. Exposure to PARP1 inhibitors is added as a qualifying criterion for MN-pCT, and methotrexate has been excluded. It is recommended that specification of the type of myeloid neoplasm is made when possible, with the appendix “post cytotoxic therapy” appended, e.g. CMML post cytotoxic therapy.

The majority of AML-pCT and MDS-pCT are associated with *TP53* mutations. The outcomes of such patients are generally worse with biallelic (multi-hit) *TP53* alterations, manifesting as ≥2 *TP53* mutations, or with concomitant 17p/*TP53* deletion or copy neutral LOH. Less frequent mutations involve genes such as *PPM1D* and DNA-damage response genes that may require additional work-up for germline predisposition.

### Myeloid neoplasms associated with germline predisposition: A novel scalable model is introduced

Myeloid neoplasms associated with germline predisposition include AML, MDS, MPN, and MDS/MPN that arise in individuals with genetic conditions associated with increased risk of myeloid malignancies. Myeloid neoplasms arising in individuals with Fanconi anemia, Down syndrome, and RASopathies are discussed in separate dedicated sections. These diseases are now classified using a formulaic approach that couples the myeloid disease phenotype with the predisposing germline genotype, e.g., AML with germline pathogenic variants in *RUNX1*. The clinical manifestations of these diseases are grouped into three subtypes under which most germline predisposition conditions can be assigned. (Table 10) Genetic counseling and evaluation of family history is an expected component of the diagnostic evaluation of index patients. Myeloid proliferations associated with Down syndrome, typically associated with somatic exon 2 or 3 *GATA1* mutation, continue to encompass two clonal conditions that arise in children with constitutional trisomy 21: transient abnormal myelopoiesis (TAM), which is confined to the first 6 months of life and myeloid leukaemia of Down syndrome (ML-DS).

**Table 10:** Subtypes of myeloid neoplasms associated with germline predisposition.

**Myeloid neoplasms with germline predisposition without a pre-existing platelet disorder or organ dysfunction**
• Germline *CEBPA* P/LP variant (CEBPA-associated familial AML)
• Germline *DDX41* P/LP variant^a^
• Germline *TP53* P/LP variant^a^ (Li-Fraumeni syndrome)
**Myeloid neoplasms with germline predisposition and pre-existing platelet disorder**
• Germline *RUNX1* P/LP variant^a^ (familial platelet disorder with associated myeloid malignancy, FPD-MM)
• Germline *ANKRD26* P/LP variant^a^ (Thrombocytopenia 2)
• Germline *ETV6* P/LP variant^a^ (Thrombocytopenia 5)
**Myeloid neoplasms with germline predisposition and potential organ dysfunction**
• Germline *GATA2* P/LP variant (GATA2-deficiency)
• Bone marrow failure syndromes
◦ Severe congenital neutropenia (SCN)
◦ Shwachman-Diamond syndrome (SDS)
◦ Fanconi anaemia (FA)
• Telomere biology disorders
• RASopathies (Neurofibromatosis type 1, CBL syndrome, Noonan syndrome or Noonan syndrome-like disorders^a,b^)
• Down syndrome^a,b^
• Germline *SAMD9* P/LP variant (MIRAGE Syndrome)
• Germline *SAMD9L* P/LP variant (SAMD9L-related Ataxia Pancytopenia Syndrome)^c^
• Biallelic germline *BLM* P/LP variant (Bloom syndrome)

^a^Lymphoid neoplasms can also occur.

^b^See respective sections.

^c^Ataxia is not always present.

*P* pathogenic, *LP* likely pathogenic.

#### Summary Box:

- Myeloid neoplasms (MDS, MDS/MPN, and AML) *post cytotoxic therapy* (MN-pCT) require full diagnostic work up; the term replaces *therapy-related*.
- Exposure to PARP1 inhibitors is added as a qualifying criterion for MN-pCT.
- The diagnostic framework for myeloid neoplasm associated with germline predisposition is restructured along a scalable model that can accommodate future refinement and discoveries.

## Myeloid/lymphoid neoplasms with eosinophilia and tyrosine kinase gene fusions

*Myeloid/lymphoid neoplasms with eosinophilia and tyrosine kinase gene fusions (MLN-TK)* are myeloid or lymphoid neoplasms driven by rearrangements involving genes encoding specific tyrosine kinases leading to fusion products in which the kinase domain is constitutively activated leading to cell signaling dysregulation that promotes proliferation and survival. (Table 11) These *BCR::ABL1*-negative diseases have long been recognized in view of their distinctive clinicopathologic features and sensitivity to TKI. They encompass a broad range of histologic types, including MPN, MDS, MDS/MPN, AML, and MPAL, as well as B- or T- lymphoblastic leukaemia/lymphoma (ALL). Extramedullary disease is common. While eosinophilia is a common and salient feature, it may be absent in some cases. From a diagnostic hierarchy standpoint, the diagnosis of MLN-TK supersedes other myeloid and lymphoid types, as well as SM. In some instances, defining genetic abnormalities of MLN-TK are acquired during course of a myeloid neoplasm such as MDS or MDS/MPN or at the time of MPN BP transformation. MLN-TK must be excluded before a diagnosis of CEL is rendered.

**Table 11:** Genetic abnormalities defining myeloid/lymphoid neoplasms with eosinophilia and tyrosine kinase gene fusions.

*PDGFRA* rearrangement
*PDGFRB* rearrangement
*FGFR1* rearrangement
*JAK2* rearrangement
*FLT3* rearrangement
*ETV6*::*ABL1* fusion
Other defined tyrosine kinase fusions:
*ETV6*::*FGFR2; ETV6*::*LYN; ETV6*::*NTRK3; RANBP2*::*ALK; BCR*::*RET; FGFR1OP*::*RET*

The majority of MLN-TK cases associated with *PDGFRA* rearrangements have cytogenetically cryptic deletion of 4q12 resulting in *FIP1L1*::*PDGFRA*, but *PDGFRA* fusions involving other partners are also identified. Cases with *PDGFRB* rearrangement result most commonly from t(5;12)(q32;p13.2) leading to *ETV6*::*PDGFRB*; however, more than 30 other partners have been identified. Cases with *FGFR1* rearrangement may manifest as chronic myeloid neoplasms or blast-phase disease of B-cell, T-cell, myeloid or mixed-phenotype origin, typically with associated eosinophilia. The characteristic cytogenetic feature is an aberration of chromosome 8p11. Detection of *JAK2* rearrangements leading to fusion products with genes other than *PCM1* have been recognized, supporting MLN-TK with *JAK2* rearrangement as a distinct type [73, 74]. Cases with *FLT3* fusion genes are particularly rare and result from rearrangements involving chromosome 13q12.2. They manifest as myeloid sarcoma with MPN features in the bone marrow or T-ALL with associated eosinophilia, but disease features and phenotypic presentation may be variable and diverse. MLN-TK with *ETV6*::*ABL1* should be separated from B-ALL with *ETV6*::*ABL1* [75].

The natural history of MLN-TK with *PDGFRA* or *PDGFRB* has been dramatically altered by TKI therapy, particularly imatinib. In contrast, patients with *FGFR1*, *JAK2* and *FLT3* fusions and *ETV6*::*ABL1* have more variable sensitivity to available newer generation TKIs [73, 76]; in most cases, long-term disease-free survival may only be achievable with allogeneic haematopoietic stem cell transplantation.

Other less common defined genetic alterations involving tyrosine kinase genes have also been discovered, and these are listed as MLN-TK subtypes under *MLN-TK with other defined tyrosine kinase fusions* until further data is accrued [77, 78].

### Summary Box:

- Family renamed myeloid/lymphoid neoplasms with eosinophilia and tyrosine kinase gene fusions (MLN-TK).
- Recognition of novel types with *JAK2* rearrangements, *FLT3* rearrangements, and *ETV6*::*ABL1* fusion.
- New scalable genetic framework introduced under MLN-TK with *other* defined tyrosine kinase fusions.

## Acute leukaemias of mixed or ambiguous lineage

Acute leukemia of ambiguous lineage (ALAL) and mixed-phenotype acute leukaemia (MPAL) are grouped under a single category in view of their overlapping clinical and immunophenotypic features, which in recent studies have been shown to also share common molecular pathogenic mechanisms. Here too, a framework for a molecular classification is laid by separating ALAL/MPAL with defining genetic abnormalities from those that are defined based on immunophenotyping only. (Table 12)

**Table 12:** Acute leukaemias of ambiguous lineage.

**Acute leukaemia of ambiguous lineage with defining genetic abnormalities**
Mixed-phenotype acute leukaemia with *BCR*::*ABL1* fusion
Mixed-phenotype acute leukaemia with *KMT2A* rearrangement
Acute leukaemia of ambiguous lineage with other defined genetic alterations
Mixed-phenotype acute leukaemia with *ZNF384* rearrangement
Acute leukaemia of ambiguous lineage with *BCL11B* rearrangement
**Acute leukaemia of ambiguous lineage, immunophenotypically defined**
Mixed-phenotype acute leukaemia, B/myeloid
Mixed-phenotype acute leukaemia, T/myeloid
Mixed-phenotype acute leukaemia, rare types
Acute leukaemia of ambiguous lineage, not otherwise specified
Acute undifferentiated leukaemia

Two new subtypes of ALAL with defining genetic alterations are added. The first subtype is MPAL with *ZNF384* rearrangement, which commonly has a B/myeloid immunophenotype and is identified in ~50% of pediatric B/myeloid MPAL with fusion partners including *TCF3*, *EP300*, *TAF15*, and *CREBBP. ZNF38*4-rearranged B/myeloid MPAL and B-ALL have similar transcriptional profile, suggesting a biological continuum [79]. The other subtype is ALAL with *BCL11B* rearrangement, which has a more heterogenous immunophenotype - identified in acute undifferentiated leukaemia (AUL) and ~20-30% of T/myeloid MPAL. *BCL11B* rearrangement is also identified in AML with minimal differentiation or without maturation and ~20-30% of ETP-ALL. [59–61, 80] These different types of acute leukaemias with stem cell, myeloid, and T-ALL features having *BCL11B* rearrangement in common suggests a biological continuum. Other genomic findings such as *PHF6* mutations and *PICALM*::*MLLT10* fusions are also enriched in MPAL, but more studies are needed.

The assignment of lineage by immunophenotyping is dependent on the strength of association between each antigen and the lineage being assessed. As a general principle, the closer the expression of an antigen is to either the intensity and/or pattern of expression seen on the most similar normal population, the more likely it reflects commitment to that lineage. For instance, variable myeloperoxidase expression with an intensity and pattern similar to that seen in early myeloid maturation is more strongly associated with myeloid lineage than uniform dim myeloperoxidase expression. In addition, demonstration of a coordinated pattern of expression of multiple antigens from the same lineage further improves the specificity of those antigens for lineage assignment, e.g. combined expression of CD19, CD22, and CD10 is more strongly associated with B lineage than each antigen individually. Given these principles, the immunophenotypic criteria to be used for lineage assignment in cases where a single lineage is not evident are revised. (Table 13)

**Table 13:** Lineage assignment criteria for mixed-phenotype acute leukaemia.

	Criterion
**B lineage**	
CD19 strong^a^	1 or more also strongly expressed: CD10, CD22, or CD79a^c^
or,	
CD19 weak^b^	2 or more also strongly expressed: CD10, CD22, or CD79a^c^
**T lineage**	
CD3 (cytoplasmic or surface)^d^	Intensity in part exceeds 50% of mature T-cells level by flow cytometry or, Immunocytochemistry positive with non-zeta chain reagent
**Myeloid lineage**	
Myeloperoxidase	Intensity in part exceeds 50% of mature neutrophil level
or,	
Monocytic differentiation	2 or more expressed: Non-specific esterase, CD11c, CD14, CD64 or lysozyme

^a^CD19 intensity in part exceeds 50% of normal B cell progenitor by flow cytometry.

^b^CD19 intensity does not exceed 50% of normal B cell progenitor by flow cytometry.

^c^Provided T lineage not under consideration, otherwise cannot use CD79a.

^d^Using anti-CD3 epsilon chain antibody.

Assessment of myeloperoxidase expression by cytochemistry and/or flow cytometry immunophenotyping plays a key role intersecting AML with minimal differentiation, T/myeloid MPAL, and ETP-ALL. Various groups have proposed flow cytometry thresholds for positive myeloperoxidase expression in acute leukaemia, ranging from 3 to 28% of blasts [81–83]. The 3% cutoff for myeloperoxidase, historically used for cytochemistry, was determined to have high sensitivity but poor specificity for general lineage assignment in acute leukaemia by flow cytometry [82, 83]. A threshold of >10% for myeloperoxidase positivity seems to improve specificity [81], but no consensus cutoff has been established.

### Summary Box:

- Acute leukaemias of mixed or ambiguous lineage are arranged into two families: ALAL with *defining genetic abnormalities* and ALAL, *immunophenotypically defined*.
- Novel genetic findings are listed as subtypes under ALAL *with other defined genetic alterations* as additional data accrues.
- Lineage assignment criteria for MPAL are refined to emphasize principles of intensity and pattern.

## Histiocytic/dendritic cell neoplasms

These neoplasms are positioned in the classification after myeloid neoplasms in recognition of their derivation from common myeloid progenitors that give rise to cells of the monocytic/histiocytic/dendritic lineages. (Table 14) Key changes in the current edition of the classification include: (1) inclusion of clonal plasmacytoid dendritic cell (pDC) diseases in this category; (2) moving follicular dendritic cell sarcoma and fibroblastic reticular cell tumor to a separate category; and, (3) addition of Rosai-Dorfman disease (RDD) and ALK-positive histiocytosis as disease types. Indeed, neoplasms that arise from lymphoid stromal cells such as follicular dendritic cell sarcoma and fibroblastic reticular cell tumor are now appropriately classified under the new chapter of “stroma-derived neoplasms of lymphoid tissues” as detailed in the companion manuscript [4].

**Table 14:** Dendritic cell and histiocytic neoplasms.

Plasmacytoid dendritic cell neoplasms
Mature plasmacytoid dendritic cell proliferation associated with myeloid neoplasm
Blastic plasmacytoid dendritic cell neoplasm
**Langerhans cell and other dendritic cell neoplasms**
*Langerhans cells neoplasms*
Langerhans cell histiocytosis
Langerhans cell sarcoma
*Other dendritic cell neoplasms*
Indeterminate dendritic cell tumour
Interdigitating dendritic cell sarcoma
**Histiocytic neoplasms**
Juvenile xanthogranuloma
Erdheim-Chester disease
Rosai-Dorfman disease
ALK-positive histiocytosis
Histiocytic sarcoma

### Plasmacytoid dendritic cell neoplasms: recognition of clonal proliferations detected in association with myeloid neoplasms and refinement/update of the diagnostic criteria for blastic plasmacytoid dendritic cell neoplasm

Mature plasmacytoid dendritic cell proliferation (MPDCP) associated with myeloid neoplasm reflects recent data showing that these represent clonal proliferation of pDCs with low grade morphology identified in the context of a defined myeloid neoplasm. Clonal MPDCP cells accumulate in the bone marrow of patients with myeloproliferative CMML harbouring activating RAS pathway mutations [84]. Patients with AML can have clonally expanded pDCs (pDC-AML), which share the same mutational landscape as CD34^+^ blasts, and frequently arise in association with *RUNX1* mutations [85, 86]. It is unknown whether the pathogenetic mechanisms leading to MPDCP in association with MDS or MDS/MPN and with AML are the same. The framework for diagnosing blastic plasmacytoid dendritic cell neoplasm remains largely the same, with emphasis on immunophenotypic diagnostic criteria. (Table 15)

**Table 15:** Immunophenotypic diagnostic criteria of blastic plasmacytoid dendritic cell neoplasm.

**Expected positive expression:**
CD123*
TCF4*
TCL1*
CD303 *
CD304*
CD4
CD56
**Expected negative markers:**
CD3
CD14
CD19
CD34
Lysozyme
Myeloperoxidase
**Immunophenotypic diagnostic criteria:**
-Expression of CD123 and one other pDC marker(*) in addition to CD4 and/or CD56.
or,
-Expression of any three pDC markers and absent expression of all expected negative markers.

### Dendritic and histiocytic neoplasms: Rosai-Dorfman disease and ALK-positive histiocytosis are new entities in the classification

Much has been learned about the molecular genetics of histiocytoses/histiocytic neoplasms in recent years. These neoplasms, in particular Langerhans cell histiocytosis/sarcoma, Erdheim-Chester disease, juvenile xanthogranuloma, RDD and histiocytic sarcoma, commonly show mutations in genes of the MAPK pathway, such as *BRAF, ARAF, MAP2K1, NRAS* and *KRAS*, albeit with highly variable frequencies, indicating a unifying genetic landscape for diverse histiocytoses and histiocytic neoplasms. ALK-positive histiocytosis furthermore converges on the MAPK pathway, which is one of the signaling pathways mediating ALK activation [87, 88]. Insights on genetic alterations have significant treatment implications, because of availability of highly effective therapy targeting components of the activated signaling pathway, such as BRAF and MEK inhibitors [88–92].

For RDD, the distinctive clinicopathologic features with accumulation of characteristic S100-positive large histiocytes showing emperipolesis, coupled with frequent gain-of-function mutations in genes of the MAPK pathway indicating a neoplastic process, provides a rationale for this inclusion and offers opportunities for targeted therapy [92–95].

ALK-positive histiocytosis, which shows a broad clinicopathologic spectrum unified by the presence of ALK gene translocation (most commonly *KIF5B*::*ALK*) and remarkable response to ALK-inhibitor therapy, has been better characterized in recent studies [88, 96]. The multisystem systemic form that typically occurs in infants, with involvement of liver, spleen and/or bone marrow, runs a protracted course but often resolves slowly, either spontaneously or with chemotherapy. Other multisystem and single-system cases occur in any age group, with involvement of two or more organs or one organ alone, respectively, most commonly central/peripheral nervous system and skin; the disease has a favourable outcome with systemic and/or surgical therapy [88, 97]. The histiocytes in ALK-positive histiocytosis can assume variable appearances including large oval cells, foamy cells and spindle cells, some with multinucleation (including Touton giant cells) or emperipolesis. That is, morphology is not entirely diagnostic, and overlaps extensively with that of juvenile xanthogranuloma and rarely RDD. Thus, it is recommended that ALK immunostaining be performed for histiocytic proliferations not conforming to defined entities, to screen for possible ALK-positive histiocytosis.

In most circumstances, classification of a dendritic cell/macrophage neoplasm as Langerhans cell histiocytosis/sarcoma, indeterminate dendritic cell tumor, interdigitating dendritic cell sarcoma or histiocytic sarcoma is straightforward. Nonetheless, there are rare cases that show overlap or hybrid features, defying precise classification [98, 99].

Among histiocytic neoplasms, a subset of cases occurs in association with or follow a preceding lymphoma/leukaemia, most commonly follicular lymphoma, chronic lymphocytic leukaemia and T- or B-ALL [100]. Since these histiocytic neoplasms usually exhibit the same clonal markers and/or hallmark genetic changes as the associated lymphoma/leukaemia, a “transdifferentiation” mechanism has been proposed to explain the phenomenon [99–101]. Furthermore, the histiocytic neoplasm and associated lymphoma/leukaemia often show additional genetic alterations exclusive to each component, suggesting that divergent differentiation or transdifferentiation occurs from a common lymphoid progenitor clone [100, 102, 103]. Histiocytoses are also sometimes associated with myeloproliferative neoplasms [104], sharing mutations with CD34^+^ myeloid progenitors [105], and with CH [106].

#### Summary Box:

- Histiocytic/dendritic cell neoplasms are regrouped and positioned to follow myeloid neoplasms in the classification scheme in view of their close ontogenic derivation.
- Mature pDC proliferation is redefined with an emphasis on recent data demonstrating shared clonality with underlying myeloid neoplasms. This framework is bound to evolve in future editions.
- Diagnostic criteria of BPDCN are refined.
- ALK-positive histiocytosis is introduced as a new entity.

## Genetic tumor syndromes with predisposition to myeloid neoplasia

Fanconi anaemia is a heterogeneous disorder caused by germline variants in the BRCA-Fanconi DNA repair pathway (≥21 genes) resulting in chromosomal breakage and hypersensitivity to crosslinking agents used for diagnosis. Clinical features include congenital anomalies, bone marrow failure, and cancer predisposition [107]. The new classification distinguishes 5 haematologic categories depending on blast percentage, cytopenia and chromosomal abnormalities [108]. Dysgranulopoiesis and dysmegakaryopoiesis are histologic indicators of progression [109]. Allogenic haematopoietic stem cell transplantation is efficacious.

The term RASopathies encompasses a diverse group of complex, multi-system disorders associated with variants in genes involved in the RAS mitogen-activating protein kinase (MAPK) pathway. Myeloid neoplasms in RASopathies involve MAPK hyperactivation, leading to myeloid cell proliferation [110]. Genomic analysis of *NF1*, *NRAS*, *KRAS*, *PTPN11*, and *CBL* from myeloid neoplasms of patients suspected of having a RASopathy is important and aids in the diagnosis of JMML in the majority of cases [111, 112]. Diagnostic criteria include pathogenic variants in genes associated with the RAS pathway and/or classic phenotype suggestive of a RASopathy [113].

## Disclaimer

The content of this article represents the personal views of the authors and does not represent the views of the authors’ employers and associated institutions. This work is intended to provide a preview and summary of content whose copyright belongs solely to the International Agency for Research on Cancer/World Health Organization. Any or all portions of the material in this work may appear in future International Agency for Research on Cancer/World Health Organization publications.

## Supplementary information

Table S1

## References

[1] Uttley L, Indave BI, Hyde C, White V, Lokuhetty D, Cree I (2020). Invited commentary-WHO Classification of Tumours: How should tumors be classified? Expert consensus, systematic reviews or both?. Int J Cancer.

[2] Salto-Tellez M, Cree IA (2019). Cancer taxonomy: pathology beyond pathology. Eur J Cancer.

[3] Cree I. The WHO Classification of Haematolymphoid Tumours. Leukemia. 2022;36:in press (same issue). 10.1038/s41375-022-01625-x PMC9252902 35732830

[4] Alaggio R, Amador C, Anagnostopoulos I, Attygalle AD, Barreto de Oliveira Araujo I, Berti E, et al. The 5th edition of the World Health Organization Classification of Haematolymphoid Tumours: Lymphoid Neoplasms. Leukemia. 2022;36:in press (same issue). 10.1038/s41375-022-01620-2 PMC9214472 35732829

[5] Bruford EA, Antonescu CR, Carroll AJ, Chinnaiyan A, Cree IA, Cross NCP (2021). HUGO Gene Nomenclature Committee (HGNC) recommendations for the designation of gene fusions. Leukemia.

[6] Jaiswal S, Fontanillas P, Flannick J, Manning A, Grauman PV, Mar BG (2014). Age-related clonal hematopoiesis associated with adverse outcomes. N Engl J Med.

[7] Beck DB, Ferrada MA, Sikora KA, Ombrello AK, Collins JC, Pei W (2020). Somatic mutations in UBA1 and severe adult-onset autoinflammatory disease. N Engl J Med.

[8] Steensma DP, Bejar R, Jaiswal S, Lindsley RC, Sekeres MA, Hasserjian RP (2015). Clonal hematopoiesis of indeterminate potential and its distinction from myelodysplastic syndromes. Blood.

[9] Greenberg PL, Tuechler H, Schanz J, Sanz G, Garcia-Manero G, Sole F (2016). Cytopenia levels for aiding establishment of the diagnosis of myelodysplastic syndromes. Blood.

[10] Hochhaus A, Larson RA, Guilhot F, Radich JP, Branford S, Hughes TP (2017). Long-term outcomes of imatinib treatment for chronic myeloid leukemia. N Engl J Med.

[11] Kalmanti L, Saussele S, Lauseker M, Müller MC, Dietz CT, Heinrich L (2015). Safety and efficacy of imatinib in CML over a period of 10 years: data from the randomized CML-study IV. Leukemia.

[12] Wang W, Cortes JE, Tang G, Khoury JD, Wang S, Bueso-Ramos CE (2016). Risk stratification of chromosomal abnormalities in chronic myelogenous leukemia in the era of tyrosine kinase inhibitor therapy. Blood.

[13] Soverini S, Bavaro L, De Benedittis C, Martelli M, Iurlo A, Orofino N, et al. Prospective assessment of NGS-detectable mutations in CML patients with nonoptimal response: the NEXT-in-CML study. Blood. 2020;135:534–41. Erratum in Blood. 2022;139:1601. 10.1182/blood.2019002969 31877211

[14] Guglielmelli P, Pacilli A, Rotunno G, Rumi E, Rosti V, Delaini F (2017). Presentation and outcome of patients with 2016 WHO diagnosis of prefibrotic and overt primary myelofibrosis. Blood.

[15] Rumi E, Boveri E, Bellini M, Pietra D, Ferretti VV, Sant’Antonio E (2017). Clinical course and outcome of essential thrombocythemia and prefibrotic myelofibrosis according to the revised WHO 2016 diagnostic criteria. Oncotarget.

[16] Barbui T, Thiele J, Passamonti F, Rumi E, Boveri E, Ruggeri M (2011). Survival and disease progression in essential thrombocythemia are significantly influenced by accurate morphologic diagnosis: an international study. J Clin Oncol.

[17] Szuber N, Finke CM, Lasho TL, Elliott MA, Hanson CA, Pardanani A (2018). CSF3R-mutated chronic neutrophilic leukemia: long-term outcome in 19 consecutive patients and risk model for survival. Blood Cancer J.

[18] Pardanani A, Lasho TL, Laborde RR, Elliott M, Hanson CA, Knudson RA (2013). CSF3R T618I is a highly prevalent and specific mutation in chronic neutrophilic leukemia. Leukemia.

[19] Pardanani A, Lasho T, Wassie E, Finke C, Zblewski D, Hanson CA (2016). Predictors of survival in WHO-defined hypereosinophilic syndrome and idiopathic hypereosinophilia and the role of next-generation sequencing. Leukemia.

[20] Cross NCP, Hoade Y, Tapper WJ, Carreno-Tarragona G, Fanelli T, Jawhar M (2019). Recurrent activating STAT5B N642H mutation in myeloid neoplasms with eosinophilia. Leukemia.

[21] Wang SA, Hasserjian RP, Tam W, Tsai AG, Geyer JT, George TI (2017). Bone marrow morphology is a strong discriminator between chronic eosinophilic leukemia, not otherwise specified and reactive idiopathic hypereosinophilic syndrome. Haematologica.

[22] Fang H, Ketterling RP, Hanson CA, Pardanani A, Kurtin PJ, Chen D (2018). A test utilization approach to the diagnostic workup of isolated eosinophilia in otherwise morphologically unremarkable bone marrow: a single institutional experience. Am J Clin Pathol.

[23] Valent P, Akin C, Gleixner KV, Sperr WR, Reiter A, Arock M, et al. Multidisciplinary challenges in mastocytosis and how to address with personalized medicine approaches. Int J Mol Sci. 2019;20:2976. 10.3390/ijms20122976 PMC6627900 31216696

[24] Reiter A, George TI, Gotlib J (2020). New developments in diagnosis, prognostication, and treatment of advanced systemic mastocytosis. Blood.

[25] Valent P, Akin C, Hartmann K, Alvarez-Twose I, Brockow K, Hermine O (2021). Updated diagnostic criteria and classification of mast cell disorders: a consensus proposal. Hemasphere.

[26] Alvarez-Twose I, Jara-Acevedo M, Morgado JM, Garcia-Montero A, Sanchez-Munoz L, Teodosio C (2016). Clinical, immunophenotypic, and molecular characteristics of well-differentiated systemic mastocytosis. J Allergy Clin Immunol.

[27] Greenberg PL, Tuechler H, Schanz J, Sanz G, Garcia-Manero G, Sole F (2012). Revised international prognostic scoring system for myelodysplastic syndromes. Blood.

[28] Malcovati L, Stevenson K, Papaemmanuil E, Neuberg D, Bejar R, Boultwood J (2020). SF3B1-mutant MDS as a distinct disease subtype: a proposal from the International Working Group for the Prognosis of MDS. Blood.

[29] Bernard E, Nannya Y, Hasserjian RP, Devlin SM, Tuechler H, Medina-Martinez JS (2020). Implications of TP53 allelic state for genome stability, clinical presentation and outcomes in myelodysplastic syndromes. Nat Med.

[30] Haase D, Stevenson KE, Neuberg D, Maciejewski JP, Nazha A, Sekeres MA (2019). TP53 mutation status divides myelodysplastic syndromes with complex karyotypes into distinct prognostic subgroups. Leukemia.

[31] Grob T, Al Hinai ASA, Sanders MA, Kavelaars FG, Rijken M, Gradowska PL (2022). Molecular characterization of mutant TP53 acute myeloid leukemia and high-risk myelodysplastic syndrome. Blood.

[32] Haferlach T, Nagata Y, Grossmann V, Okuno Y, Bacher U, Nagae G (2014). Landscape of genetic lesions in 944 patients with myelodysplastic syndromes. Leukemia.

[33] Tashakori M, Kadia TM, Loghavi S, Daver NG, Kanagal-Shamanna R, Pierce SR, et al. TP53 copy number and protein expression inform mutation status across risk categories in acute myeloid leukemia. Blood. 2022, in press. 10.1182/blood.2021013983 PMC9346958 35390143

[34] Yoshizato T, Dumitriu B, Hosokawa K, Makishima H, Yoshida K, Townsley D (2015). Somatic mutations and clonal hematopoiesis in aplastic anemia. N Engl J Med.

[35] Nazha A, Seastone D, Radivoyevitch T, Przychodzen B, Carraway HE, Patel BJ (2015). Genomic patterns associated with hypoplastic compared to hyperplastic myelodysplastic syndromes. Haematologica.

[36] Fattizzo B, Ireland R, Dunlop A, Yallop D, Kassam S, Large J (2021). Clinical and prognostic significance of small paroxysmal nocturnal hemoglobinuria clones in myelodysplastic syndrome and aplastic anemia. Leukemia.

[37] Estey E, Hasserjian RP, Dohner H (2022). Distinguishing AML from MDS: a fixed blast percentage may no longer be optimal. Blood.

[38] DiNardo CD, Garcia-Manero G, Kantarjian HM (2022). Time to blur the blast boundaries. Cancer.

[39] Chen X, Fromm JR, Naresh KN (2022). “Blasts” in myeloid neoplasms - how do we define blasts and how do we incorporate them into diagnostic schema moving forward?. Leukemia.

[40] Pastor V, Hirabayashi S, Karow A, Wehrle J, Kozyra EJ, Nienhold R (2017). Mutational landscape in children with myelodysplastic syndromes is distinct from adults: specific somatic drivers and novel germline variants. Leukemia.

[41] Schwartz JR, Ma J, Lamprecht T, Walsh M, Wang S, Bryant V (2017). The genomic landscape of pediatric myelodysplastic syndromes. Nat Commun.

[42] Baumann I, Fuhrer M, Behrendt S, Campr V, Csomor J, Furlan I (2012). Morphological differentiation of severe aplastic anaemia from hypocellular refractory cytopenia of childhood: reproducibility of histopathological diagnostic criteria. Histopathology.

[43] Sahoo SS, Pastor VB, Goodings C, Voss RK, Kozyra EJ, Szvetnik A (2021). Clinical evolution, genetic landscape and trajectories of clonal hematopoiesis in SAMD9/SAMD9L syndromes. Nat Med.

[44] Sahoo SS, Kozyra EJ, Wlodarski MW (2020). Germline predisposition in myeloid neoplasms: Unique genetic and clinical features of GATA2 deficiency and SAMD9/SAMD9L syndromes. Best Pract Res Clin Haematol.

[45] Montalban-Bravo G, Kanagal-Shamanna R, Guerra V, Ramos-Perez J, Hammond D, Shilpa P (2021). Clinical outcomes and influence of mutation clonal dominance in oligomonocytic and classical chronic myelomonocytic leukemia. Am J Hematol.

[46] Calvo X, Garcia-Gisbert N, Parraga I, Gibert J, Florensa L, Andrade-Campos M (2020). Oligomonocytic and overt chronic myelomonocytic leukemia show similar clinical, genomic, and immunophenotypic features. Blood Adv.

[47] Geyer JT, Tam W, Liu YC, Chen Z, Wang SA, Bueso-Ramos C (2017). Oligomonocytic chronic myelomonocytic leukemia (chronic myelomonocytic leukemia without absolute monocytosis) displays a similar clinicopathologic and mutational profile to classical chronic myelomonocytic leukemia. Mod Pathol.

[48] Patnaik MM, Timm MM, Vallapureddy R, Lasho TL, Ketterling RP, Gangat N (2017). Flow cytometry based monocyte subset analysis accurately distinguishes chronic myelomonocytic leukemia from myeloproliferative neoplasms with associated monocytosis. Blood Cancer J.

[49] Selimoglu-Buet D, Wagner-Ballon O, Saada V, Bardet V, Itzykson R, Bencheikh L (2015). Characteristic repartition of monocyte subsets as a diagnostic signature of chronic myelomonocytic leukemia. Blood.

[50] Cargo C, Cullen M, Taylor J, Short M, Glover P, Van Hoppe S (2019). The use of targeted sequencing and flow cytometry to identify patients with a clinically significant monocytosis. Blood.

[51] Carr RM, Vorobyev D, Lasho T, Marks DL, Tolosa EJ, Vedder A (2021). RAS mutations drive proliferative chronic myelomonocytic leukemia via a KMT2A-PLK1 axis. Nat Commun.

[52] Xicoy B, Triguero A, Such E, Garcia O, Jimenez MJ, Arnan M (2018). The division of chronic myelomonocytic leukemia (CMML)-1 into CMML-0 and CMML-1 according to 2016 World Health Organization (WHO) classification has no impact in outcome in a large series of patients from the Spanish group of MDS. Leuk Res.

[53] Loghavi S, Sui D, Wei P, Garcia-Manero G, Pierce S, Routbort MJ (2018). Validation of the 2017 revision of the WHO chronic myelomonocytic leukemia categories. Blood Adv.

[54] Quintana-Bustamante O, Lan-Lan Smith S, Griessinger E, Reyal Y, Vargaftig J, Lister TA (2012). Overexpression of wild-type or mutants forms of CEBPA alter normal human hematopoiesis. Leukemia.

[55] Wen XM, Hu JB, Yang J, Qian W, Yao DM, Deng ZQ (2015). CEBPA methylation and mutation in myelodysplastic syndrome. Med Oncol.

[56] Gao Y, Jia M, Mao Y, Cai H, Jiang X, Cao X (2022). Distinct mutation landscapes between acute myeloid leukemia with myelodysplasia-related changes and de novo acute myeloid leukemia. Am J Clin Pathol.

[57] Lindsley RC, Mar BG, Mazzola E, Grauman PV, Shareef S, Allen SL (2015). Acute myeloid leukemia ontogeny is defined by distinct somatic mutations. Blood.

[58] Padella A, Simonetti G, Paciello G, Giotopoulos G, Baldazzi C, Righi S, et al. Novel and rare fusion transcripts involving transcription factors and tumor suppressor genes in acute myeloid leukemia. Cancers. 2019;11:1951. 10.3390/cancers11121951 PMC6966504 31817495

[59] Wang W, Beird H, Kroll CJ, Hu S, Bueso-Ramos CE, Fang H (2020). T(6;14)(q25;q32) involves BCL11B and is highly associated with mixed-phenotype acute leukemia, T/myeloid. Leukemia.

[60] Di Giacomo D, La Starza R, Gorello P, Pellanera F, Kalender Atak Z, De Keersmaecker K (2021). 14q32 rearrangements deregulating BCL11B mark a distinct subgroup of T-lymphoid and myeloid immature acute leukemia. Blood.

[61] Montefiori LE, Bendig S, Gu Z, Chen X, Polonen P, Ma X, et al. Enhancer hijacking drives oncogenic BCL11B expression in lineage-ambiguous stem cell leukemia. Cancer Discov. 2021;11:2846–67. 10.1158/2159-8290.CD-21-0145 PMC8563395 34103329

[62] Liu W, Hasserjian RP, Hu Y, Zhang L, Miranda RN, Medeiros LJ (2011). Pure erythroid leukemia: a reassessment of the entity using the 2008 World Health Organization classification. Mod Pathol.

[63] Wang SA, Hasserjian RP (2015). Acute erythroleukemias, acute megakaryoblastic leukemias, and reactive mimics: a guide to a number of perplexing entities. Am J Clin Pathol.

[64] Montalban-Bravo G, Benton CB, Wang SA, Ravandi F, Kadia T, Cortes J (2017). More than 1 TP53 abnormality is a dominant characteristic of pure erythroid leukemia. Blood.

[65] Wang W, Wang SA, Medeiros LJ, Khoury JD (2017). Pure erythroid leukemia. Am J Hematol.

[66] Mazzella FM, Smith D, Horn P, Cotelingam JD, Rector JT, Shrit MA (2006). Prognostic significance of pronormoblasts in erythrocyte predominant myelodysplastic patients. Am J Hematol.

[67] Kowal-Vern A, Cotelingam J, Schumacher HR (1992). The prognostic significance of proerythroblasts in acute erythroleukemia. Am J Clin Pathol.

[68] Werstein B, Dunlap J, Cascio MJ, Ohgami RS, Fan G, Press R (2020). Molecular discordance between myeloid sarcomas and concurrent bone marrows occurs in actionable genes and is associated with worse overall survival. J Mol Diagn.

[69] Greenland NY, Van Ziffle JA, Liu YC, Qi Z, Prakash S, Wang L (2021). Genomic analysis in myeloid sarcoma and comparison with paired acute myeloid leukemia. Hum Pathol.

[70] Engel NW, Reinert J, Borchert NM, Panagiota V, Gabdoulline R, Thol F (2021). Newly diagnosed isolated myeloid sarcoma-paired NGS panel analysis of extramedullary tumor and bone marrow. Ann Hematol.

[71] Takahashi K, Wang F, Kantarjian H, Doss D, Khanna K, Thompson E (2017). Preleukaemic clonal haemopoiesis and risk of therapy-related myeloid neoplasms: a case-control study. Lancet Oncol.

[72] Kuendgen A, Nomdedeu M, Tuechler H, Garcia-Manero G, Komrokji RS, Sekeres MA (2021). Therapy-related myelodysplastic syndromes deserve specific diagnostic sub-classification and risk-stratification-an approach to classification of patients with t-MDS. Leukemia.

[73] Schwaab J, Naumann N, Luebke J, Jawhar M, Somervaille TCP, Williams MS (2020). Response to tyrosine kinase inhibitors in myeloid neoplasms associated with PCM1-JAK2, BCR-JAK2 and ETV6-ABL1 fusion genes. Am J Hematol.

[74] Tang G, Sydney Sir Philip JK, Weinberg O, Tam W, Sadigh S, Lake JI (2019). Hematopoietic neoplasms with 9p24/JAK2 rearrangement: a multicenter study. Mod Pathol.

[75] Yao J, Xu L, Aypar U, Meyerson HJ, Londono D, Gao Q (2021). Myeloid/lymphoid neoplasms with eosinophilia/ basophilia and ETV6-ABL1 fusion: cell-of-origin and response to tyrosine kinase inhibition. Haematologica.

[76] Chen JA, Hou Y, Roskin KM, Arber DA, Bangs CD, Baughn LB (2021). Lymphoid blast transformation in an MPN with BCR-JAK2 treated with ruxolitinib: putative mechanisms of resistance. Blood Adv.

[77] Carll T, Patel A, Derman B, Hyjek E, Lager A, Wanjari P (2020). Diagnosis and treatment of mixed phenotype (T-myeloid/lymphoid) acute leukemia with novel ETV6-FGFR2 rearrangement. Blood Adv.

[78] Telford N, Alexander S, McGinn OJ, Williams M, Wood KM, Bloor A (2016). Myeloproliferative neoplasm with eosinophilia and T-lymphoblastic lymphoma with ETV6-LYN gene fusion. Blood Cancer J.

[79] Alexander TB, Gu Z, Iacobucci I, Dickerson K, Choi JK, Xu B (2018). The genetic basis and cell of origin of mixed phenotype acute leukaemia. Nature.

[80] Montefiori LE, Mullighan CG (2021). Redefining the biological basis of lineage-ambiguous leukemia through genomics: BCL11B deregulation in acute leukemias of ambiguous lineage. Best Pract Res Clin Haematol.

[81] van den Ancker W, Westers TM, de Leeuw DC, van der Veeken YF, Loonen A, van Beckhoven E (2013). A threshold of 10% for myeloperoxidase by flow cytometry is valid to classify acute leukemia of ambiguous and myeloid origin. Cytometry B Clin Cytom.

[82] Guy J, Antony-Debre I, Benayoun E, Arnoux I, Fossat C, Le Garff-Tavernier M (2013). Flow cytometry thresholds of myeloperoxidase detection to discriminate between acute lymphoblastic or myeloblastic leukaemia. Br J Haematol.

[83] Bras AE, Osmani Z, de Haas V, Jongen-Lavrencic M, Te Marvelde JG, Zwaan CM (2021). Standardised immunophenotypic analysis of myeloperoxidase in acute leukaemia. Br J Haematol.

[84] Lucas N, Duchmann M, Rameau P, Noel F, Michea P, Saada V (2019). Biology and prognostic impact of clonal plasmacytoid dendritic cells in chronic myelomonocytic leukemia. Leukemia.

[85] Zalmai L, Viailly PJ, Biichle S, Cheok M, Soret L, Angelot-Delettre F (2021). Plasmacytoid dendritic cells proliferation associated with acute myeloid leukemia: phenotype profile and mutation landscape. Haematologica.

[86] Xiao W, Chan A, Waarts MR, Mishra T, Liu Y, Cai SF (2021). Plasmacytoid dendritic cell expansion defines a distinct subset of RUNX1-mutated acute myeloid leukemia. Blood.

[87] Jaffe ES, Chan JKC (2022). Histiocytoses converge through common pathways. Blood.

[88] Kemps PG, Picarsic J, Durham BH, Helias-Rodzewicz Z, Hiemcke-Jiwa L, van den Bos C (2022). ALK-positive histiocytosis: a new clinicopathologic spectrum highlighting neurologic involvement and responses to ALK inhibition. Blood.

[89] McClain KL, Bigenwald C, Collin M, Haroche J, Marsh RA, Merad M (2021). Histiocytic disorders. Nat Rev Dis Primers.

[90] Emile JF, Cohen-Aubart F, Collin M, Fraitag S, Idbaih A, Abdel-Wahab O (2021). Histiocytosis. Lancet.

[91] Salama HA, Jazieh AR, Alhejazi AY, Absi A, Alshieban S, Alzahrani M (2021). Highlights of the management of adult histiocytic disorders: langerhans cell histiocytosis, erdheim-chester disease, rosai-dorfman disease, and hemophagocytic lymphohistiocytosis. Clin Lymphoma Myeloma Leuk.

[92] Diamond EL, Durham BH, Ulaner GA, Drill E, Buthorn J, Ki M (2019). Efficacy of MEK inhibition in patients with histiocytic neoplasms. Nature.

[93] Chakraborty R, Abdel-Wahab O, Durham BH. MAP-kinase-driven hematopoietic neoplasms: a decade of progress in the molecular age. Cold Spring Harb Perspect Med. 2021;11:a034892. 10.1101/cshperspect.a034892 PMC7770072 32601132

[94] Durham BH, Lopez Rodrigo E, Picarsic J, Abramson D, Rotemberg V, De Munck S (2019). Activating mutations in CSF1R and additional receptor tyrosine kinases in histiocytic neoplasms. Nat Med.

[95] Jacobsen E, Shanmugam V, Jagannathan J (2017). Rosai-Dorfman disease with activating KRAS mutation - response to Cobimetinib. N Engl J Med.

[96] Chang KTE, Tay AZE, Kuick CH, Chen H, Algar E, Taubenheim N (2019). ALK-positive histiocytosis: an expanded clinicopathologic spectrum and frequent presence of KIF5B-ALK fusion. Mod Pathol.

[97] Chan JK, Lamant L, Algar E, Delsol G, Tsang WY, Lee KC (2008). ALK+ histiocytosis: a novel type of systemic histiocytic proliferative disorder of early infancy. Blood.

[98] Emile JF, Abla O, Fraitag S, Horne A, Haroche J, Donadieu J (2016). Revised classification of histiocytoses and neoplasms of the macrophage-dendritic cell lineages. Blood.

[99] Feldman AL, Arber DA, Pittaluga S, Martinez A, Burke JS, Raffeld M (2008). Clonally related follicular lymphomas and histiocytic/dendritic cell sarcomas: evidence for transdifferentiation of the follicular lymphoma clone. Blood.

[100] Egan C, Lack J, Skarshaug S, Pham TA, Abdullaev Z, Xi L (2021). The mutational landscape of histiocytic sarcoma associated with lymphoid malignancy. Mod Pathol.

[101] Shao H, Xi L, Raffeld M, Feldman AL, Ketterling RP, Knudson R (2011). Clonally related histiocytic/dendritic cell sarcoma and chronic lymphocytic leukemia/small lymphocytic lymphoma: a study of seven cases. Mod Pathol.

[102] Pericart S, Waysse C, Siegfried A, Struski S, Delabesse E, Laurent C (2020). Subsequent development of histiocytic sarcoma and follicular lymphoma: cytogenetics and next-generation sequencing analyses provide evidence for transdifferentiation of early common lymphoid precursor-a case report and review of literature. Virchows Arch.

[103] Brunner P, Rufle A, Dirnhofer S, Lohri A, Willi N, Cathomas G (2014). Follicular lymphoma transformation into histiocytic sarcoma: indications for a common neoplastic progenitor. Leukemia.

[104] Papo M, Diamond EL, Cohen-Aubart F, Emile JF, Roos-Weil D, Gupta N (2017). High prevalence of myeloid neoplasms in adults with non-Langerhans cell histiocytosis. Blood.

[105] Durham BH, Roos-Weil D, Baillou C, Cohen-Aubart F, Yoshimi A, Miyara M (2017). Functional evidence for derivation of systemic histiocytic neoplasms from hematopoietic stem/progenitor cells. Blood.

[106] Cohen Aubart F, Roos-Weil D, Armand M, Marceau-Renaut A, Emile JF, Duployez N (2021). High frequency of clonal hematopoiesis in Erdheim-Chester disease. Blood.

[107] Dutzmann CM, Spix C, Popp I, Kaiser M, Erdmann F, Erlacher M (2022). Cancer in children with fanconi anemia and ataxia-telangiectasia-a nationwide register-based cohort study in Germany. J Clin Oncol.

[108] Behrens YL, Göhring G, Bawadi R, Coktu S, Reimer C, Hoffmann B (2021). A novel classification of hematologic conditions in patients with Fanconi anemia. Haematologica.

[109] Cioc AM, Wagner JE, MacMillan ML, DeFor T, Hirsch B (2010). Diagnosis of myelodysplastic syndrome among a cohort of 119 patients with fanconi anemia: morphologic and cytogenetic characteristics. Am J Clin Pathol.

[110] Gaipa G, Bugarin C, Cianci P, Sarno J, Bonaccorso P, Biondi A (2015). Peripheral blood cells from children with RASopathies show enhanced spontaneous colonies growth in vitro and hyperactive RAS signaling. Blood Cancer J.

[111] Kim HS, Lee JW, Kang D, Yu H, Kim Y, Kang H (2021). Characteristics of RAS pathway mutations in juvenile myelomonocytic leukaemia: a single-institution study from Korea. Br J Haematol.

[112] Stieglitz E, Taylor-Weiner AN, Chang TY, Gelston LC, Wang YD, Mazor T (2015). The genomic landscape of juvenile myelomonocytic leukemia. Nat Genet.

[113] Bhoj EJ, Yu Z, Guan Q, Ahrens-Nicklas R, Cao K, Luo M (2017). Phenotypic predictors and final diagnoses in patients referred for RASopathy testing by targeted next-generation sequencing. Genet Med.

