# Supplementary material for: The 5th edition of the World Health Organization Classification of Haematolymphoid Tumours: Myeloid and Histiocytic/Dendritic Neoplasms
Source: Leukemia. 2022 Jun 22;36(7):1703–19. doi: 10.1038/s41375-022-01613-1 (PMC9252913; doi:10.1038/s41375-022-01613-1)
Supplement: Supplementary file 1 — Table S1 [file 41375_2022_1613_MOESM1_ESM.docx]

**SUPPLEMENTAL DATA**

**Table S1**. Examples of clonal haematopoiesis driver mutations (listed in approximate order of frequency).

|  |  |  |
| --- | --- | --- |
| **Gene name** | **Criteria for classification as a CH driver mutation** | **Reference transcript** |
| **Common and/or clinically significant mutations** | | |
| DNMT3A | Frameshift/nonsense/splice-site; Missense in aa range: p.292-350, 482-614 and 634-912 | NM_022552 |
| TET2 | Frameshift/nonsense/splice-site; Missense in aa range: p.1104-1481 and 1843-2002 | NM_001127208 |
| ASXL1 | Frameshift/nonsense/splice-site in exon 11-12 | NM_015338 |
| JAK2 | V617F; Missense/indel in aa range: p.536-547 | NM_004972 |
| TP53 | Frameshift/nonsense/splice-site; Missense in aa range: p.72, 95-288 and 337 | NM_001126112 |
| SF3B1 | Missense in terminal HEAT domains (p.529-1201) | NM_012433 |
| PPM1D | Frameshift/nonsense/splice-site in exon 5/6 | NM_003620 |
| SRSF2 | Missense/in-frame deletion involving P95 | NM_003016 |
| ZBTB33 | Missense involving functional domains | NM_001184742 |
| IDH1 | Missense at R132 | NM_005896 |
| IDH2 | Missense at R140 / R172 | NM_002168 |
| U2AF1 | Missense at S34 / R156 / Q157 | NM_006758 |
| KRAS | Missense at G12 / G13 / Q61 / A146 | NM_033360 |
| NRAS | Missense at G12 / G13 / Q61 | NM_002524 |
| CTCF | Frameshift/nonsense/splice-site, R377C, R377H, P378A, P378L | NM_006565 |
| CBL | Missense in Linker/RING finger domains (p.345-434) | NM_005188 |
| GNB1 | Missense at K57 / G53 / I81 | NM_002074 |
| BRCC3 | Frameshift/nonsense/splice-site | NM_024332 |
| PTPN11 | Missense in aa range p.58-76 and p.491-510 | NM_002834 |
| GNAS | Missense at R201 | NM_016592 |
| BCOR | Frameshift/nonsense/splice-site | NM_001123385 |
| BCORL1 | Frameshift/nonsense/splice-site | NM_021946 |
| **Other mutations** | | |
| BRAF | Missense in aa range p.590-615; Missense at G469 | NM_004333 |
| CALR | Frameshift in exon 9 | NM_004343 |
| CEBPA | Frameshift/nonsense/splice-site | NM_004364 |
| CREBBP | Frameshift/nonsense/splice-site | NM_004380 |
| CSF1R | Missense at L301 / Y969 | NM_005211 |
| CSF3R | T615A, T618I, truncating c.741-791 | NM_000760 |
| CUX1 | Frameshift/nonsense/splice-site | NM_181552 |
| ETV6 | Frameshift/nonsense/splice-site | NM_001987 |
| EZH2 | Frameshift/nonsense/splice-site; Missense in SET domain (p.617-732) | NM_001203247 |
| GATA2 | Frameshift/nonsense/splice-site, R293Q, N317H, A318T, A318V, A318G, G320D, L321P, L321F, L321V, Q328P, R330Q, R361L, L359V, A372T, R384G, R384K | NM_001145661 |
| JAK3 | M511T, M511I, A572V, A572T, A573V, R657Q, V715I, V715A | NM_000215 |
| KDM6A | Frameshift/nonsense/splice-site | NM_021140 |
| KIT | ins503, V559A, V559D, V559G, V559I, V560D, V560A, V560G, V560E, del560, E561K, del579, P627L, P627T, R634W, K642E, K642Q, V654A, V654E, H697Y, H697D, E761D, K807R, D816H, D816Y, D816F, D816I, D816V, D816H, del551-559 | NM_000222 |
| KMT2A | Frameshift/nonsense/splice-site | NM_005933 |
| MPL | S505G, S505N, S505C, L510P, del513, W515A, W515R, W515K, W515S, W515L, A519T, A519V, Y591D, W515-518KT | NM_005373 |
| MYD88 | L265P | NM_002468 |
| NOTCH1 | Frameshift/nonsense/splice-site/missense in exon 26-34 | NM_017617 |
| PHF6 | Frameshift/nonsense/splice-site | NM_001015877 |
| PIGA | Frameshift/nonsense/splice-site | NM_002641 |
| PRPF40B | Frameshift/nonsense/splice-site | NM_001031698 |
| PTEN | Frameshift/nonsense/splice-site | NM_000314 |
| RAD21 | Frameshift/nonsense/splice-site | NM_006265 |
| RUNX1 | Frameshift/nonsense/splice-site, S73F, H78Q, H78L, R80C, R80P, R80H, L85Q, P86L, P86H, S114L, D133Y, L134P, R135G, R135K, R135S, R139Q, R142S, A165V, R174Q, R177L, R177Q, A224T, D171G, D171V, D171N, R205W, R223C | NM_001001890 |
| SETBP1 | D868N, D868T, S869N, G870S, I871T, D880N, D880Q | NM_015559 |
| SF1 | Frameshift/nonsense/splice-site | NM_004630 |
| SF3A1 | Frameshift/nonsense/splice-site | NM_005877 |
| SMC1A | Missense at R96 / R586 | NM_006306 |
| SMC3 | Frameshift/nonsense/splice-site | NM_005445 |
| STAG2 | Frameshift/nonsense/splice-site | NM_006603 |
| STAT3 | Missense in SH2 domain (p.580-670) | NM_139276 |
| U2AF2 | Missense in RNA recognition motifs domains (p.149-231, p.259-337, p.381-462) | NM_007279 |
| WT1 | Frameshift/nonsense/splice-site | NM_024426 |
| ZRSR2 | Frameshift/nonsense/splice-site | NM_005089 |
